# A Sheepish Account of Ireland's Changing Role in an Imperial Age, 1780–1824 *

**DOI:** 10.1177/03324893251385227

**Published:** 2025-11-18

**Authors:** Mary O'Sullivan

**Affiliations:** 27212 Université de Genève, Switzerland

**Keywords:** improvement, sheep grazing, woollen manufactures, political economy, profit

## Abstract

This essay addresses the question of why Ireland's sheep and wool diminished in economic importance during an era in which they seemed to count so much for other states. From 1780 to 1800, the Irish Government and Parliament enjoyed greater autonomy to shape what Julian Hoppit describes as a ‘political economy of wool' but used it to discount sheep and wool. After the Union, there was a belated effort by Irish political elites to promote sheep and wool in Ireland's improvement, but it proved a spectacular failure. Integrating political economy and economic history, the essay shows the importance of wool in Ireland’s political dynamics and the profound impact of these politics on the economic history of Irish sheep and wool.

In February 1813, Napoleon's Minister of the Interior, the Comte de Montalivet, emphasised the importance of sheep in his comprehensive assessment of the economic condition of the Napoleonic Empire. Thirty-five million sheep, he explained, produced 120 million pounds of wool in France and even if higher quality wools continued to be imported, Montalivet insisted the ongoing improvement of French wool would ‘one day free us from this necessity’. That same year, a man of long-standing influence in the British Empire, John Holroyd, Lord of Sheffield, offered a similar account of its challenges and ambitions in the wool trade. His ‘principal apprehension’ was ‘whether our manufacturers will be enabled to procure an adequate supply of the raw material’, and he argued for encouraging ‘the growth and manufacture of British fine wools’, so that Britain would ‘become independent of other countries for the materials of staple manufacture’.^1^

These men's observations hint at the importance of sheep in Europe's leading empires in the late eighteenth and early nineteenth centuries. In both France and England, sheep dominated all other livestock in importance, their numbers rivalling the human population in France and reaching an extraordinary four times the number of people in England.^2^ Still, the number of sheep tells us little about the precise roles that sheep played. Raw material for woollen manufactures was crucial, of course, but sheep were more important than their wool. In fact, a breakdown of the revenues from a Norfolk sheep farm in England in the late 1780s suggests that wool ranked only third in importance, far behind its meat and even its dung!^3^

For many European ‘improvers’ in the late eighteenth and early nineteenth centuries, sheep were seen as the beating heart of a powerful economic and environmental system. The great French chemist, Antoine Lavoisier, emphasised that ‘[t]he English system of cultivation is almost the opposite’ of its French counterpart, since ‘[t]hey cultivate to raise and feed their stock’. He claimed its advantage was

evident in the considerable increase in manure and thus in the fertility of soil. You see it in cheaper meat for city dwellers and even country people. You see it in the manufactures furnished with an abundance of national output, and the producers of woollen cloth freed from dependence on foreign states for raw materials.^4^

Lavoisier was to lose his head in the French revolution, but sheep remained an important focus of French ‘improvement’ from the *Ancien Régime* through revolution and the Napoleonic era. As for Lavoisier, so for many of the French improvers who followed him, Great Britain exemplified the manifold advantages of an empire committed to sheep. The British themselves largely agreed, maintaining their long-standing prohibition on the export of their wool for more than a century and a half through to 1824. A growing body of historical literature shows there was nothing natural or accidental about the prominent roles that sheep played in Britain and France. It reveals what Julian Hoppit describes as a distinctive ‘political economy of wool’ in each of these empires, consisting of trade prohibitions, official sheep stealing and breeding programmes, as well as policies for land reform and colonial settlement.^5^

As France and Britain sought to protect and promote their sheep, they paid close attention to other states as well as each other. Besides Spain, with its renowned flocks of fine-woolled Merino sheep, it was Ireland that was seen as one of Europe's natural homes for sheep. Certainly, that was what William Petty claimed when he wrote *The Political Anatomy of Ireland* in the 1670s. He emphasised that most of Ireland's population lived in ‘very wretched’ housing, but that ‘their Cloathing [was] far better than that of the French Peasants, or the poor of most other Countreys; which advantage they have from their Wooll’. He estimated that there were four million sheep on the island growing eight million pounds of wool or 7.2 lbs per person.^6^ Nor was Petty alone in his attention to Irish sheep with other visitors to late seventeenth-century Ireland remarking on the country's natural affinity for sheep.^7^

But if sheep and wool seemed important to Ireland in Petty's time, their prominence waned during the century and a half that followed. In the writing of prominent Irish historians of earlier generations, the English Parliament's infamous Woollen Act in 1699 featured large in this story. More than thirty years ago, however, Leslie Clarkson claimed that: ‘[t]he stereotype established by Alice Murray and George O'Brien at the beginning of this century, of an industry crippled by the act of 1699, has not survived the revisionist work of the last thirty years’.^8^ A rich body of historical research on Ireland's woollen sector points to the revival of the Irish woollen sector from the middle of the eighteenth century – even before British export restrictions on Irish sheep and wool were lifted in late 1779 – and the persistence of the Irish woollen industry into the early nineteenth century.^9^ The revisionist interpretation of Irish economic history has been contested by a number of scholars, with Denis O’Hearn arguing that wool and woollens played a crucial role in the systematic ‘regional peripheralisation’ of Ireland within the British Empire.^10^ Nevertheless, there is little doubt that revisionism succeeded in re-defining how Irish economic history was written.

However, if we ask what became of Irish sheep and wool by the late 1830s, it is striking that the revisionists were led to the same fading importance of sheep and wool in Ireland as their predecessors, but without a clear explanation of that outcome. The earliest official estimate of the number of sheep in Ireland comes from the Census of 1841 and suggests that there were only 2.1 million sheep in Ireland by then, a mere quarter of a sheep for every human being.^11^ By 1838, moreover, as Louis M. Cullen observed, ‘the Irish woollen textile industry, which in the late 18th century still supplied by far the greater part of Irish consumption, supplied only about 14 per cent of Irish needs. … Even the Irish countryman, once clothed in home-produced frieze, was now dressed in imported fabric’.^12^ Revisionists pointed to a variety of problems but without offering a clear interpretation of what caused the demise of Ireland's supposedly resurgent woollen sector by the early decades of the nineteenth century.

When it comes to Irish sheep, moreover, it is remarkable how difficult they are to find in Irish historical writing. One of the most sophisticated analyses of the transformation of Irish agriculture in the late eighteenth and early nineteenth centuries comes from Kenneth Connell, the scholar this lecture honours. Yet, even Connell was to adopt a practice that pervades subsequent research on Irish agricultural history, lumping sheep with beef and pigs in an undifferentiated pasture sector. There are regional agricultural histories like Thomas Power's work on Tipperary that help us see Ireland's sheep a bit more clearly, but they inspired few successors. In a recent contribution, James Stafford seeks to re-open the question of agricultural improvement in Ireland by focussing on political debates about tillage but says hardly anything about sheep.^13^

More generally, sheep and wool have attracted few enthusiasts among Irish historians in the last thirty years as we see in reviews of publication trends in *Irish Economic and Social History*, including the most recent one by Graham Brownlow, Catherine Cox and Eoin McLaughlin.^14^ In fact, one of the most important recent contributions on the subject comes from an historian of the Norwich textile industry, Michael Nix, given his attention to Ireland's important trade in worsted yarn.^15^ Still, a major puzzle remains about what happened to Ireland's woollen sector in the decades from the American Revolution through the Napoleonic wars. In this essay, therefore, I ask why Ireland's sheep and wool diminished in economic importance during an era in which they seemed to count so much not only in Europe's leading empires, but in smaller states like Saxony, not to mention more distant territories? To address it, the essay is divided into two periods that marked distinct phases in the political economy of Irish wool, each of which had crucial and surprising implications for wool growth and manufacture.

With the lifting of British export restrictions on the Irish woollen sector in 1779, followed by legislative independence in 1782, the Kingdom of Ireland enjoyed greater autonomy from 1780 to 1800 in determining its political economy of wool. However, as we shall see, the Irish Government and Parliament used their political power to discount the importance of the growth and manufacture of wool, substantially undermining the roles they played in the kingdom's economy. The second period runs from 1801, when the Act of Union brought Irish sheep and wool within the ambit of Britain's political economy of wool, to 1824 when long-standing restrictions on the British wool market were lifted. Following the Union, sheep and wool witnessed a belated but determined effort by Irish political elites to restore sheep as a focus of their nation's improvement, but their ‘sheepish enlightenment’ culminated in spectacularly poor results. The Irish woollen-manufacturing sector enjoyed an ambitious but brief revival before fading to smaller proportions from the mid-1820s.

## Discounting Wool and Sheep in the Kingdom of Ireland, 1780–1800

Getting hold of some of Spain's protected Merino sheep was a focal point of other European states’ efforts to improve their sheep and wool. Britain was a latecomer in this regard when Joseph Banks, the famous naturalist and explorer, launched its Merino programme in the late 1780s. Thus, Irish-born Lord Sheffield put Banks in touch with William Burton Conyngham of Slane Castle in Co. Meath, who had already introduced Merino sheep to Ireland. Conyngham had secured six Spanish Merinos through clandestine channels in 1784 and explained to Banks that he hoped: ‘I shall live to see some advantage accrue to Ireland from my undertakings’ in the promotion of cultivation and commerce.^16^ Elsewhere in Ireland, however, there was growing concern about the economic potential of wool as the basis for Irish improvement.

In lifting its restrictions on Irish exports in late 1779, Britain had responded to growing demands from Ireland for ‘free trade’.^17^ In a letter to the British Government in the summer of 1779, the acting chancellor of the Irish Exchequer, John Foster, argued:

[t]hat the Common Strength, Wealth and Commerce of both Kingdoms require new, speedy and effectual Sources of Wealth to be opened to this Country, and that it is the Interest of Britain either considered separately or as the Head of the British Empire to open those Sources by a Removal of all Restrictions on our Trades.

As far as specific restrictions were concerned, Foster identified ‘those on Woolen, as the most oppressive’, explaining that ‘[i]t is our natural Manufacture, so much so, that the Restraints of near a Century have not been able to divert this Kingdom from the breed of Sheep which as far as I can judge is not diminished…’.^18^

Woollen and worsted cloth had been manufactured in Ireland throughout the eighteenth century, but while export restrictions were in place, it was sold exclusively in the domestic market, with important manufacturing centres including Dublin, Carrick-on-Suir and Cork. Moreover, even though prohibitive duties excluded Irish woollen goods from the British market, low protective duties in Ireland meant that the kingdom's woollen manufactures were exposed to significant competition from British manufacturers even in the Irish market.^19^ And as the British sought to deal with the damaging effects of the American Revolution and war on their exports, they increased their Irish sales.

**Figure 1.**
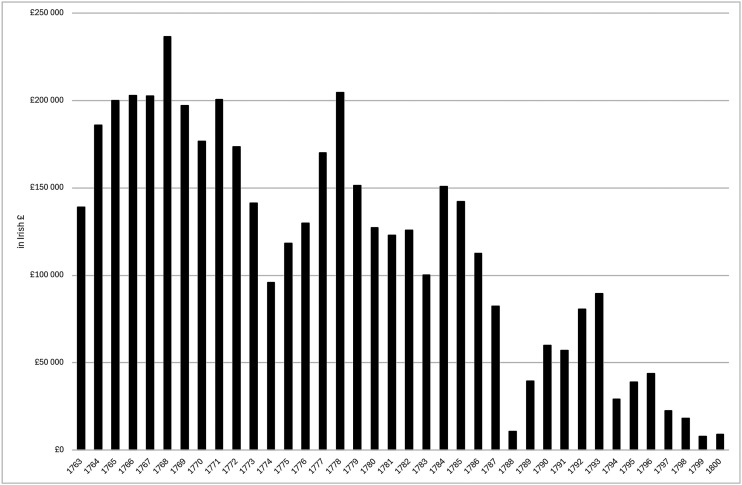
Irish exports of worsted yarn, 1763–1800. *Source*: *Ledgers of Imports and Exports, Ireland*, CUST 15, various issues, National Archives, Kew Gardens, London.

At about the same time, there was a major downturn in the only export trade that Ireland had successfully established in wool. When the British made an exception to its export restrictions for woollen and worsted yarn, a major trade developed in Irish worsted or ‘bay’ yarn to England.^20^ It was conducted largely by Quaker merchants in Ireland who sold yarn to English buyers, with Norwich merchants playing a particularly important role in this regard.^21^ The export industry reached a peak in quantity terms of 2.8 million lbs of yarn in the late 1760s, the equivalent of approximately 3.7 million lbs of raw wool.^22^ However, since the trade in worsted yarn depended on the economic fortunes of British worsted manufactures, it faced a marked decline in the unsettled conditions of the early 1770s, then a brief rebound before a major downturn from the late 1770s through the end of the American war (see Figure 1).

**Figure 2.**
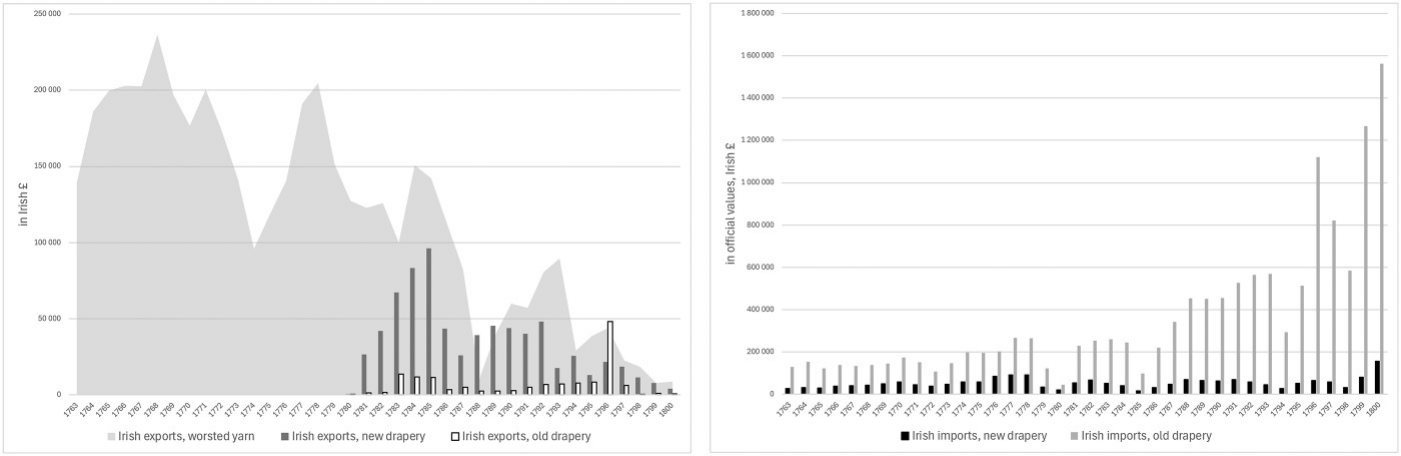
Irish trade in woollen and worsted goods, 1763–1800. *Source*: *Ledgers of Imports and Exports, Ireland*, CUST 15, various issues, National Archives, Kew Gardens, London.

These developments contributed to a broader economic downturn in Ireland in the late 1770s, encouraging non-importation agreements across Ireland and eventually pressure from the Irish Parliament for British trade concessions. When they were awarded in late 1779, there was elation in Ireland and optimism that a brighter future for the Irish woollen sector would lead economic recovery. However, the benefits of the trade concessions proved to be disappointing for Ireland's woollen sector with the expansion in exports of worsted and woollen cloth barely compensating for the decline in Irish exports of worsted yarn over the same period. Moreover, the lifting of non-importation agreements led to a revival in woollen and worsted imports from Britain, which surpassed the total value of exports by the Irish woollen sector by early 1783 (Figure 2).^23^

A further political crisis ensued in Ireland marked by social protest and a flood of petitions to the Irish Parliament. The woollen industry played a prominent role with calls for restrictions on exports of vital materials and protection of the Irish market. Under growing pressure, the House of Commons established a committee in October 1783 to consider ‘what measures may be proper for the improvement of the manufactures of this kingdom’. Led by Luke Gardiner, Member of Parliament for Dublin, it conducted the most detailed investigation ever undertaken into the state of Irish manufactures. The Gardiner committee concluded that without protection, Ireland's woollen industry and other so-called ‘infant’ manufactures could not survive in the face of British competition.^24^

### The Woollen Sector's Profits in Inexorable Decline, 1780–1800

The Gardiner committee examined many Irish manufacturers and merchants from the woollen and worsted trades. Some had given testimony in previous enquiries when they focussed on the purportedly excessive wages and insubordinate behaviour of Dublin weavers. In the Gardiner investigation, in contrast, their primary concern was the cost of raw materials, specifically the high price of Irish wool compared with English wool. Witnesses explained that three quarters of the wool produced in Ireland was combing wool to make worsted yarn and cloth. They emphasised that both the export of worsted yarn and the manufacture of worsted cloth were under severe pressure due to the high price of Irish combing wool compared with its English counterpart. The gap between wool prices could persist due to Britain’s longstanding ban on exports of its relatively inexpensive wool, even to its sister kingdom.

The relatively high price of combing wool had struck Arthur Young when he toured Ireland in the mid-1770s. He explained that Irish prices had pulled away from English wool prices from mid-century to almost double their levels by the late 1760s. By then, as Michael Nix shows, Norwich merchants were expressing concern about the high prices that the Irish yarn merchants were paying for wool.^25^ Unfortunately, there is no existing series of Irish wool prices, with even the extensive research on Irish prices by Peter Solar and Liam Kennedy excluding wool.^26^ However, by drawing on evidence from Young's *Tour*, parliamentary papers as well as newspapers, it is possible to build a price series for Irish combing wool that can be compared with prices of English combing wool of similar quality.

**Figure 3.**
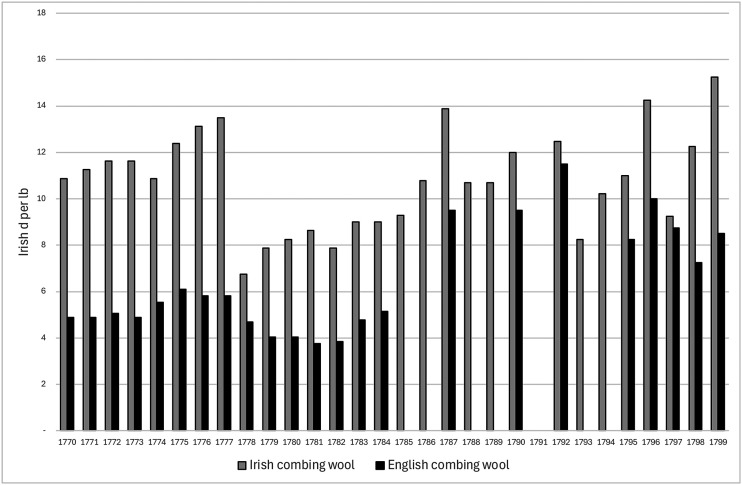
Estimated prices of Irish and English wool, 1764–1800. *Source*: Arthur Young, *A Tour in Ireland with General Observations on the Present State of that Kingdom Made in the Years 1776, 1777, and 1778 and Brought Down to the Year 1779* (Dublin, 1780); *Report of the Lords of the Committee of Council Appointed for the Consideration of all Matters Relating to Trade and Foreign Plantations Upon the Two Questions Referred to them by His Majesty's Order in Council*, 14 January 1785 (London, 1785), pp. 27–8, Great Britain Parliament. House of Commons; *Dublin Evening Post & Saunders's News-Lette*r, various issues.

As Figure 3 shows, Irish combing wool was about double the price of English combing wool through the 1770s, peaking in 1777. A sharp decline followed, but then a rapid rebound, and since English prices for combing wool took longer to recover from their own decline, the premium paid for Irish combing wool re-appeared. Therefore, it is not hard to understand why witnesses in the Gardiner investigation were so concerned about the high price of Irish combing wool. As significant as what they said in this regard, is the evidence they provided on the impact of Ireland's expensive combing wool for the manufacturing costs of Irish worsted yarn and cloth.

**Figure 4.**
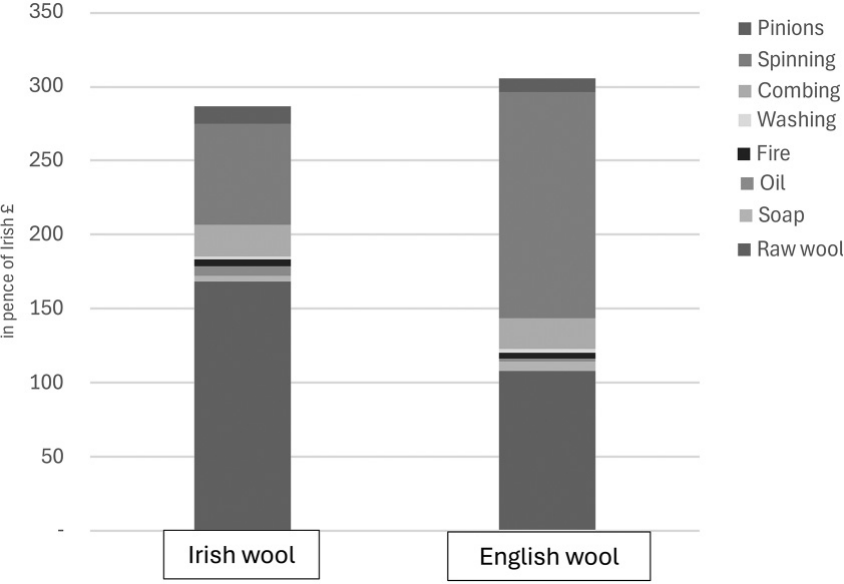
Costs of producing worsted yarn, similar quality, Irish and English yarn (1 stone of raw wool). *Source*: Testimony of Benjamin Haughton, Manufacturer of New Drapery, Dyer & Finisher, Gardiner, *Report*, cxlvi.

It was widely understood that Ireland's worsted yarn was competitive on the English market because the low wages paid to rural female spinners compensated for the high prices of wool in Ireland. Joshua Pim was a prominent Irish merchant in worsted yarn and he had explained to an earlier Irish parliamentary committee that: ‘a woman can earn in a whole day, but 2 1/2 d’ spinning worsted yarn in Ireland, which was half of what English spinsters were paid.^27^ The evidence furnished in the Gardiner investigation allows us to be more precise about the economic characteristics of Ireland's export trade in worsted yarn. Benjamin Haughton, an Irish manufacturer of worsted cloth, offered a detailed breakdown of the costs of producing worsted yarn of a similar quality from a stone of raw wool in both Ireland and England. As Figure 4 shows, spinning was not the only labour needed to produce worsted yarn since the combing of long wool, typically performed by men, preceded spinning. However, Haughton's estimates show that combing accounted for only a small proportion of the total cost of producing worsted yarn and that there was no appreciable difference in the cost of combing labour in Ireland and England.

In contrast, his estimates show how important spinning was in determining the total cost of worsted yarn. Just as Pim had said, Irish women earned only half what their English counterparts did for spinning a similar quality of worsted yarn from a stone of raw wool. Thus, Haughton's estimates show exactly what it meant for the low wages of thousands of women spinners in rural Ireland to compensate for high wool prices paid to Irish sheep grazers. But since the spinning earnings of Irish women were already low, there was limited scope for lowering them much further as Irish combing wool prices continued to rise.

**Figure 5.**
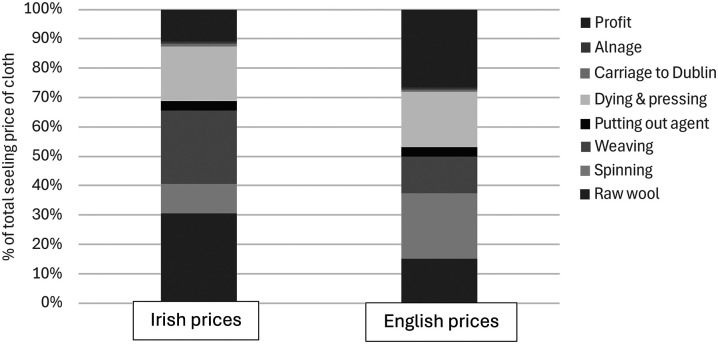
Profits and costs of Irish manufacture of worsted cloth. Estimated percentage of selling price of cloth of a Durant cloth, 30 yards, broad stuff, weighs about 9 lb, based on Irish versus English prices of labour and materials. *Source*: Various testimonies of worsted manufacturers, ‘Report from the Grand Committee of Trade’, 6 March 1782; testimony of Benjamin Haughton, Gardiner, *Report*, cxlvi.

The decline in Irish exports of worsted yarn was not the only fallout from increasing prices of Irish combing wool. With the removal of trade restrictions, the manufacture of worsted cloth had given greatest cause for optimism about the future of Irish ‘infant manufactures’ but Irish combing wool was an essential input for worsted manufactures. Evidence submitted to the Gardiner investigation allows us to see, as Figure 5 shows, that it would have been much more profitable to make worsted cloth in Ireland if English prices were paid for inputs instead of Irish prices. One reason was that the wages of weavers were higher in Ireland, amounting to twenty-five per cent of the selling price of cloth compared to 12.5 per cent at English prices, which Luke Gardiner attributed in an earlier memorandum to ‘the dearness of House rent and provisions’ and ‘the frequent Combinations of Journeymen’.^28^ More important, however, was the fact that raw materials were so expensive in Ireland, representing 41 per cent of the selling price at Irish wool prices compared to a hypothetical 21 per cent at English prices. Overall, Irish worsted cloth was manufactured at a relative cost disadvantage that amounted to 32.5 per cent of the selling price of the cloth when Irish prices were paid, with 61 per cent of the disadvantage stemming from the higher price of Irish raw wool.

Even in the absence of political restrictions, therefore, the economic characteristics of Irish worsted manufactures limited their export prospects. They were excluded by prohibitive tariffs from the British market, but the threat of British competition was a major one in any foreign market where Irish manufactures could hope to sell. Sure enough, Irish worsted manufactures reported that they had little export presence. Thus, they remained largely dependent on their home market but even there the high wool prices they paid, and Dublin's relatively high wages for weavers, exposed Irish manufactures to increasing competition from rising British imports.

For cheaper worsteds, transport costs offered some protection, as did the small duty of 2*d* per yard on Irish imports of worsted cloth. Still, by the time of the Gardiner investigation, the continued rise in wool prices was squeezing the margin of profits even on cheaper worsted manufactures. The situation was worse for higher value worsted cloth since the duty provided little protection and British imports were rising in this segment. Even more concerning, as Figure 2 suggests, were the large imports from Britain of woollen cloth or old drapery made from carded wool.

Woollen manufactures had traditionally been strong in Ireland due to a significant supply of native-grown wool from short-wool sheep that thrived in mountainous terrain. Nevertheless, by the time of the Gardiner investigation, the Irish manufacture of woollen cloth was characterised as ‘almost totally extinct’.^29^ Again, raw wool was the main problem with the quality and cost of carding wool as major concerns. Given Irish sheep graziers’ strong orientation towards combing wool, carding wool was harder to find and, worse still, as an Irish wool manufacturer explained: ‘the clothier was necessitated to pay the price, which combers made, and that too for wool not fit for his purpose, when, at the same time, in England, the clothing wool rated from three to four shillings a stone under the price given here’.^30^ The low quality of most Irish carding wool meant that it was unsuitable for finer woollens, which depended on imports of Spanish Merino wool. However, Irish manufacturers of finer woollens did not have the option of mixing expensive Spanish Merino with finer qualities of native wool to lower their costs, as their counterparts in the west of England did, making them vulnerable to imports of finer qualities of woollen cloth from Britain.

Based on the previous analysis, it is clear why working people in Ireland's worsted and woollen industries thought they were doomed unless political steps were taken to address the root of their problems. In substantiating their complaints about raw wool, the Gardiner investigation placed the Irish Parliament on the horns of a political dilemma. It concluded with a recommendation for higher protective tariffs to allow Irish worsted and woollen manufactures to survive the onslaught of British competition given the high cost of their raw materials. Yet, even before the Gardiner plan was made public, it ran into formidable opposition due to a concerted effort within the Irish Government to discount woollen manufactures in the Kingdom of Ireland.

In an internal memorandum in early 1784, senior government official, John Parnell, claimed that: ‘[m]uch greater consequence has been given to the woolen trade of Ireland, than it seems to merit’.^31^ He was a close confidant of John Foster, by then Irish Chancellor of the Exchequer, who echoed the same view and justified it in terms of Irish agricultural conditions: ‘The woollen business does not promise ever to be extensive. The moistness of our climate gives a better return from land in tillage and black cattle than in sheep’.^32^ Despite his earlier characterisation of wool as Ireland's ‘natural manufacture’, Foster now emphasised the limited prospects of sheep in a country undergoing major agricultural change.^33^

Foster was a convincing speaker, with greater command of economic facts than most of his parliamentary interlocutors, and he succeeded in undermining support for the Gardiner report when it was discussed in parliament in April 1784. Besides his objection to wool growing as the basis for Irish agricultural improvement, Foster echoed Gardiner's earlier memorandum in pointing to the concentration of Irish wool manufacture in the city of Dublin. It employed large numbers of journeymen there, from a variety of religious backgrounds, but Foster emphasised that manufacturing in the capital city drove up costs, encouraged a lack of discipline and fostered social disorder.^34^

Foster's attack on the Gardiner report was a parliamentary triumph but there was such a powerful backlash in the streets and newspapers of Dublin that there were fears for the stability of the Kingdom of Ireland and its place in the British Empire. The Chancellor acted quickly to stifle the protests, wielding several powerful sticks but adding a new carrot as well. He announced a bounty scheme in May 1784 to put Ireland's woollen sector back to work with a substantial bounty on all domestic or export sales of woollen and worsted cloth.^35^

**Figure 6.**
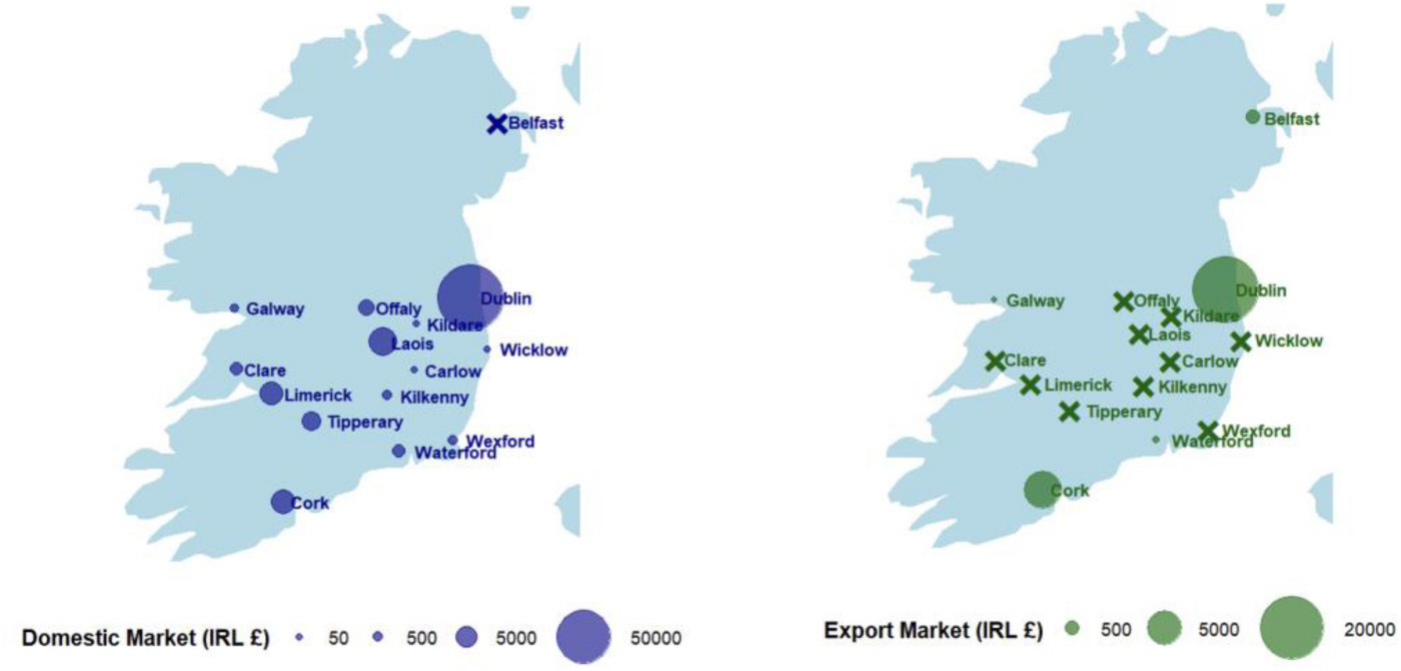
Mapping Irish woollen and worsted production. Value of manufactures reported for May–November 1784. x on the two maps designates small centres of manufacture, circles show relative scale of larger ones. *Source*: Author's analysis based on Foster, *Report on Bounties*, maps by Gaia Valenti.

The returns from that scheme, reported in February 1785, are used in Figure 6 to offer a snapshot of the scale and distribution of Ireland's commercial woollen sector that completes what we know from existing research.^36^ It confirmed that by the mid-1780s, the production and exports of Ireland's woollen sector was dominated by worsted cloth, with woollen manufactures accounting for only ten per cent of total production. Moreover, as Figure 6 shows, the overwhelming dominance of the capital city as a manufacturing centre stands out both for the domestic and export market. It encouraged Foster to greater effort to break Dublin's dominance, by introducing new bounties that he made conditional on moving manufactures to regions that were ten miles or more outside of Dublin.^37^

By then, Foster was heavily embroiled in bringing William Pitt the Younger's Irish commercial propositions to fruition. Pitt's so-called free trade agreement with Ireland could have had significant implications for the country's woollen sector had it implied the liberalisation of trade in raw materials as well as finished goods. However, the long-standing prohibition on exports of English wool was written into one of the propositions. Pitt offered the following explanation of its inclusion to Ireland's Lord Lieutenant: ‘In the great article of the woollen, if we confine the raw material to ourselves, and let Ireland do the same, perhaps the produce of Ireland, and what she can import from other places, can never enable her to supplant us to a great extent in this article’.^38^

From the perspective of the Irish woollen sector, Pitt's commercial propositions had little to commend them since they offered no respite from the high cost of its raw materials. Luke Gardiner was forthright in this regard in a letter to the Chief Secretary of Ireland, Thomas Orde. ‘I must beg leave to say a few words on the subject of the raw material’, Gardiner wrote, since he was of the opinion that ‘a free intercourse of export and import of wool, woollen and bay yarns would be highly beneficial to this Country and would not injure England’. He insisted that: ‘[w]e can never expect to make an advantage of this manufacture as long as the price of the material is held up at so high a rate’. But he pointed out that ‘[t]he landed men here of the South and West will object to it, because the natural consequence will be the lowering of the price of wool in Ireland’.^39^

With or without Pitt's propositions, therefore, the economic prospects of the Irish woollen industry were inextricably linked to the supply of wool from Irish graziers. Mr Nixon, a Dublin manufacturer, acknowledged in a pamphlet addressed to Ireland's noblemen, gentlemen, and farmers in 1784 that there might be ‘some degree of curiosity and surprize’ that ‘a mechanic should thus address a grazier’. But he explained that graziers ‘may be said to be the manufacturers’ of Irish wool and that it was ‘of more general importance, that it may be made more perfect from their hands than it is’ for ‘the multitudes, who depend on it for support’ since ‘their skill can be of little effect’ without improvement in the wool of Ireland.^40^

There is little sign that Irish graziers took note of Irish woollen manufacturers’ complaints about the low quality and high prices of Irish wool. In a discussion of the Eden–Rayneval treaty in the Irish Parliament, and the easier access to the French market that it would imply for Irish woollen and worsted manufactures, one member observed that ‘our wool is at present at an enormous price, a price never known here before’, rendering it impossible for Irish manufacturers to compete in France. Another added that ‘wool is out of all proportion dear in Ireland, at not less than twenty-one shillings the stone at this day’.^41^ The unprecedented price of Irish wool had already damaged Ireland's export trade in bay yarn. It had shifted towards Cork, as yarn merchants tried to compensate with cheaper spinning in remoter parts of the country, but exports of worsted yarn lost ground after a brief recovery in the mid-1780s to low levels in the late 1780s and early 1790s under the pressure of high wool prices (Figure 2).^42^

Weakening demand for Irish wool exerted some downward pressure on its price in 1788, as Figure 3 shows, but it recovered until a more definitive check in 1793. There were reports from the great fair in Ballinasloe that substantial quantities of wool were left unsold as buyers refused to buy and prices fell sharply. In a petition to the Irish Parliament in 1793, Dublin's manufacturers of worsted yarn and cloth explained that they were ‘in great distress and reduced to the lowest ebb of penury and famine, owing to the general stagnation and almost total annihilation which have of late taken place in the various branches of manufacture’. They noted that:

[i]n the year 1789 two thousand looms were employed in the vicinity of Dublin on the aforesaid fabrics, which gave employ to several thousands of both sexes, but from the above period the aforesaid number of looms have gradually diminished to less than one-fourth and our brethren in the interior parts of the province of Leinster, and also the province of Munster, have felt the effects of the general stagnation.^43^

They added that ‘in the course of the last two months the material, wool and bay yarn, has fallen in price twenty per cent, which must exceedingly injure the wool-grower and landed proprietor at the ensuing wool fairs, and also the wool-comber and manufacturers of bay-yarn’.^44^

From that year, however, the Irish Parliament offered indirect support to Irish wool growing and manufacture in a series of acts to defray the charge of clothing its newly created and rapidly expanding Irish militia.^45^ Employment in Dublin's woollen sector rebounded from the ‘great distress’ of 1792 as ‘[m]any contracts were given for this purpose [army clothing] to the manufacturers of the Liberties, and great benefits derived from them’.^46^ Nevertheless, the closing years of the Kingdom of Ireland were difficult for textile manufactures in the capital city. In early 1798, when mass arrests were made in Dublin on leaked news of the planned rebellion, working men from the city's textile trades were by far the largest group represented among the 1,000 people arrested.^47^ Besides Dublin's woollen manufactures, substantial activity was undertaken in and around Cork in the 1790s; by 1800, 457 looms were still operating there but, as Bielenberg notes, that was much lower than the estimated 2,000 in the Cork area at mid-century. Across Ireland, he observes, ‘employment in all the major centres of the industry had declined by the end of the eighteenth century’.^48^

Military demand allowed the price of Irish wool to return to record-breaking levels in the second half of the 1790s even if internal disorder and rebellion in Ireland made the price unstable in the late 1790s. But Ireland's high wool prices made it extremely difficult for its woollen and worsted manufacturers to survive as commercial operations. By the end of the eighteenth century, Irish manufactures had no foreign presence since exports of worsted cloth and yarn had collapsed to negligible levels by the Act of Union. In parallel, as Figure 2 shows, growing quantities of British goods were imported to meet Irish commercial demand, despite the barrier of Irish protective duties. By the turn of the century, even imports of new drapery were increasing, though it was old drapery or woollens that still constituted the vast majority of Irish imports.

The fact that British imports were so successful in woollen manufactures reflects the significance of short wool in their cost structure and the relative disadvantage that implied for Irish manufacturers. The example of blanket manufactures in Kilkenny makes it clear that despite multiple initiatives – including the adoption of water-powered spinning jennies, a reduction in the amount of wool used for each blanket, and an increase in their selling price – the cost of raw wool still rose from sixty-three to seventy per cent of manufacturing costs between 1782 and 1800. As one Kilkenny manufacturer concluded: ‘Home consumption only being supplied by us, very little would be done, not even the looms kept at work, did not Government give a decided preference to Irish blanketing for the army use at present’.^49^ As the Kingdom of Ireland ended its days, therefore, the Irish manufacturers of woollen and worsted cloth that survived were the ones that catered to military demand.^50^ That begs the question of why Irish graziers repeatedly allowed prices to go so high that they undermined manufacturing demand for their own wool? In addressing it, we turn to the conditions that governed the Irish growth of sheep's wool.

### The Elusive Profits from Irish Sheep, 1780–1800

Even well-informed observers blamed the high prices and low quality of Irish wool on the fact that there was such a ready market for raw wool from France and for worsted yarn from England. However, these explanations lack credibility both from the mid-1780s to the early 1790s, and again in the late 1790s as the price of Irish wool rebounded to reach unprecedented levels. Both of these periods of high wool prices coincided with plummeting exports of bay yarn and negligible exports of raw wool to France. An alternative explanation of the high price of Irish wool was proposed by Lord Sheffield based on a comparison of the profit potential of Irish sheep graziers compared with their English counterparts.^51^

Notwithstanding the ‘supposed extraordinary value’ of Irish wool in the mid-1780s, Sheffield observed that ‘the quantity of sheep is said to decrease in Ireland, and, undoubtedly, would decrease much more if the price was as low as in England’.^52^ The apparent paradox was explained, Sheffield believed, by differences in how English and Irish graziers made profits from sheep. He emphasised that the market for mutton in Ireland was extremely limited and haughtily predicted that it would not grow until Irish manufacturers ‘are more industrious, and consequently can afford to live better and consume more meat’. Until then, a high price was necessary to ensure an adequate supply of Irish wool since ‘sheep, when wool is low, will not answer as well as in England, where the price of mutton is much higher, and makes it answer to the farmer to raise sheep when the price of wool would not’.^53^ As Sheffield implied, the only market for mutton for Irish sheep graziers was the domestic market, reflecting the fact that mutton was not shipped long distances at the time, which accounts for its absence from Irish export statistics in contrast to substantial exports of beef and pork.^54^

In Ireland, the primary markets for mutton were in the larger cities like Cork and especially Dublin. The largest flocks of Irish sheep were bred in the lowlands of Connaught, notably in the counties of Roscommon, Tipperary and Galway. Some of them were sold at the Ballinasloe fair to Leinster graziers who fattened them in Meath and other counties close to Dublin. Sheep graziers like these Connacht and Leinster graziers with indirect and direct links to Irish mutton markets, could count on making some of their profits from meat as well as wool. For most of the sheep in Ireland, however, the country's limited mutton consumption meant that the profits they generated had to come mainly from wool.

Sheffield pointed to a further feature of the Irish agricultural system that made high wool prices more necessary in Ireland than in England to encourage sheep grazing. He explained that even though more of the South Downs of Sussex were ploughed than before, ‘[a]rtificial grasses, rape, turneps [*sic*], and other improvements in husbandry, enable the farmers to keep larger stocks’. Evoking the English system of cultivation with sheep at its core that his French contemporary, Antoine Lavoisier, so admired, Sheffield contrasted the complementarity between tillage and grazing in England with a trade-off in Ireland. He believed: ‘[i]t will require great improvements in the whole system of agriculture… before she can considerably increase her sheep without decreasing her tillage’. Until then, Sheffield said, sheep grazing and tillage would be seen as alternatives and he insisted that tillage was ‘of much more consequence to Ireland’.^55^

There is ample evidence that Irish sheep grazing was organised differently from its English counterparts. For a variety of reasons, as agricultural historians have emphasised, Ireland's largest sheep graziers functioned more like ranchers than farmers. As a result, mixed farming with sheep at its core was much less common than in England and concentrated mainly on gentlemen's estates where agricultural improvement was consciously pursued along English lines. Nevertheless, Irish historians have contested Sheffield's related claim of a necessary trade-off in Ireland between sheep grazing and tillage.

Such a trade-off had troubled Arthur Young when he had travelled through Ireland ten years earlier and he attributed it to Ireland's bounties on the inland carriage of corn. These bounties were justified explicitly by the Irish Parliament in the 1750s as a means of promoting tillage in Ireland's leading sheep grazing counties, but Young was horrified by what he saw as their effects.^56^ Less than a decade later, as Sheffield well knew, the Irish Parliament had gone even further in this direction: the Corn Law of 1784 was so closely associated with Sheffield's good friend, John Foster, that it was referred to as ‘Foster's Corn Law’. In emphasising the greater consequence for Ireland of tillage over sheep, therefore, Sheffield was alluding to Foster's law and predicting it would promote tillage at the expense of sheep grazing.

Assessing such claims of a trade-off between grazing and tillage in Ireland from the late 1750s is a complex task as Kenneth Connell pointed out in his classic book, *The Population of Ireland*. He contended that there was a widespread movement from pasture to tillage from the 1780s to the 1830s, especially in Ireland's grazing counties. He attributed it to the bounties on inland carriage of corn from 1757 to 1798, the export bounties and import restrictions offered by Foster's Corn Law from 1784 to 1806, and the rising prices of corn from 1784 to 1815. Nevertheless, he said it was ‘open to doubt’ that the shift ‘diminished the country's yield of pastoral products’, invoking the buoyant prices and exports of butter, pork and bacon to support his point. Rather than good grass being ploughed to make bad tillage, as Young argued, Connell suggested that the expansion of tillage, especially in the grazing counties, must have come from ploughing up ‘much land formerly waste, or grazing of very low productivity’.^57^

**Figure 7.**
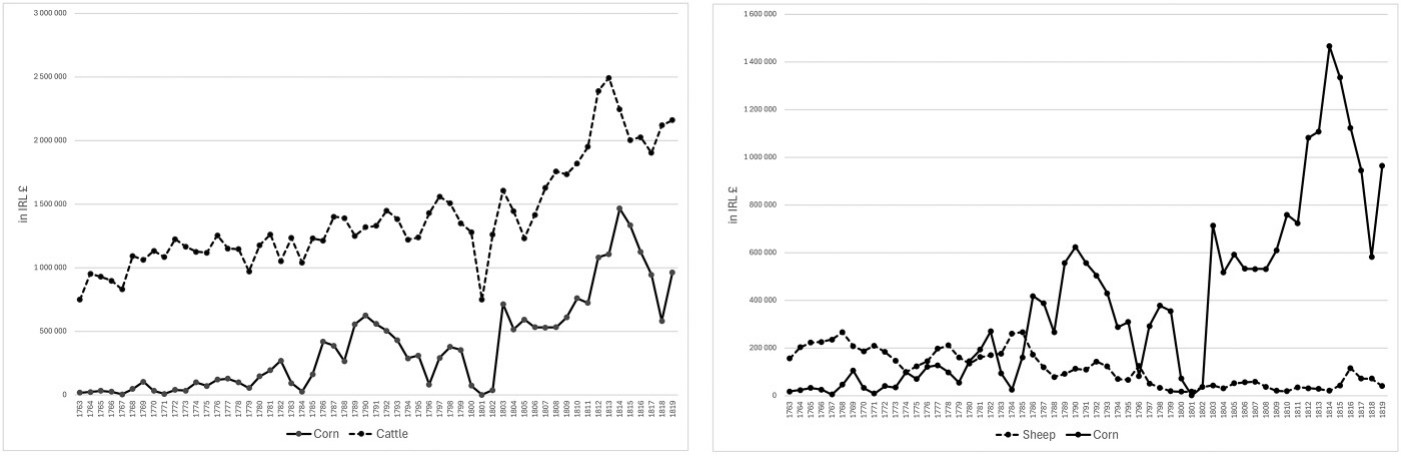
Irish exports of corn versus pastoral products, 1763–1819. *Source*: *Ledgers of Imports and Exports, Ireland*, CUST 15, various issues, National Archives, Kew Gardens, London.

Other Irish historians have reached similar conclusions with the additional benefit of Peter Solar's research on the history of prices to support them. Like Connell, they focus on cattle, taking exports of beef and butter as proxies for pastoral exports, which they compare with exports of corn. Sure enough, as Figure 7 shows, a systematic comparison based on Ireland's trade statistics shows little evidence of a trade-off as exports of corn expanded from the mid-1770s. However, if we compare exports of sheep-based products with corn exports, as shown in Figure 7, we get a different impression with 1784 marking a turning point in the emergence of an apparent trade-off.

Suggestive as export trends might seem, they offer an inadequate basis for evaluating whether sheep grazing lost out to tillage in Ireland in the late eighteenth century. An analysis of the export bounties created by Foster's Corn Law in 1784 allows us to estimate the premiums on an acre of tillage when Irish prices satisfied the thresholds established for export bounties. But the crucial question is how these premiums compared to the profits from grazing sheep and that depended, as Sheffield explained, on whether graziers sold wool or mutton and wool.

Graziers who sold mutton benefitted from the fact that prices rose sharply with the passage of Foster's Corn Law, then doubled and nearly tripled by the Act of Union. None of the grains produced in Ireland enjoyed a similar expansion in their market prices and the export bounties paid were not large enough to alter the balance in favour of tillage.^58^ Still, even if selling mutton became highly attractive as the price tripled, the numbers of sheep sold at the Ballinasloe fair hovered around a stable average of 66,000. There was a brief spike just before the Act of Union when mutton prices reached ‘extravagant rates’ but when large numbers of sheep went unsold at these prices, they diminished.^59^ What is clear, therefore, is that rearing sheep for mutton was a lucrative business for some Irish graziers but there seem to have been limits on how many sheep could be sold for their meat in Ireland, just as Sheffield argued.

For other sheep graziers in Ireland, wool was their main source of revenue and their economic calculus looked quite different. The price of Irish wool did rise sharply following Foster's Corn Law, as we have seen, but Irish manufacturers proved to be more price sensitive than the country's mutton consumers. Thus, the price dynamics of Irish wool were less attractive than for mutton with a ‘mere’ doubling of Irish wool prices between the early 1780s and 1800. Even more important, graziers selling wool earned much lower revenues than graziers who sold mutton and wool, making the revenues from corn including export bounties relatively more attractive to them.^60^

In his *Statistical Survey of the County of Meath*, published in 1802, Robert Thompson offered evidence to show the importance of this distinction. Meath was a grazing county that was closely tied to the mutton market in Dublin. For that reason, as Thompson observed, sheep were sent by the Connacht graziers to their counterparts in Meath to be fattened there for Dublin consumers.^61^ Each Meath grazier, he noted, selected sheep at the Ballinasloe fair based on the quality of the pasture available to him, and then kept them ‘as well as possible until spring, at which time they are sold in Dublin, at a considerable profit’.^62^

Thompson illustrated this point with estimates for a tenant farmer in Meath in 1800–01, which are shown in simplified form in Table 1 to show the profit from buying a ewe for 16*s* at the Ballinasloe fair in October 1800. The results suggest the overwhelming importance of mutton in generating the ‘considerable profit’ that Thompson evoked, with wool representing a mere 7 per cent of total revenues. The cost estimates make no allowance for land rental but Thompson dealt with that subject elsewhere in his survey. He offered a range of 5*s* to 50*s* for an acre for land in Meath, explaining that the lower rents were available only on older leases and that 30*s* an acre was a reasonable average for the whole county.^63^ If we assume that four sheep – two ewes and their lambs – could be fattened on one acre of land, land rent can be added to the calculation in Table 1 at 15*s*. That would lower the estimated profits for Thompson's sheep grazier, and a finer-grained analysis would add further costs. Still, it is difficult to see how anything other than a ‘considerable profit’ would be generated on the sale of mutton and wool. If only wool was sold, in contrast, the calculations highlight the much lower profits to be generated; even if a farmer could raise the 16*s* to buy a ewe, he would not be able to pay land rent of 15*s* with 5*s* of wool.

**Table 1.** Profits from Sheep for a Tenant Farmer in Meath.

Category	£ s d	% of total revenues
Prime cost (purchase of ewe)	£0 16*s* 0*d*	20
Lamb sold	£1 5*s* 0*d*	32
Four pounds of wool	£0 5*s* 0*d*	7
Ewe sold	£2 8*s* 0*d*	61
Total revenues	£3 18*s* 6*d*	100
Profit	£3 2*s* 6*d*	80

*Source*: Thompson, *Meath*, pp. 225–6.

It was this unattractive calculus that confronted Irish sheep farmers who did not enjoy as favourable an access to mutton markets as Meath graziers. Indeed, it caught Arthur Young's attention when he toured Ireland and encountered some of the leading sheep graziers in Connacht. He explained that he ‘wanted much to be informed of their profit, but it is exceedingly difficult to come near it, for not a grazier in the country but denies his making any thing considerable’. He noted ‘this is supposed to be a great piece of art’ but acknowledged he was ‘apt to think the truth not so far from the declaration, at least as well as I am able to judge from the information I have received’. Moreover, he suggested that the soil of ‘exceptional fertility’ would ‘do very well for tillage’ and ‘pay infinitely better’, although the landlord would ‘suffer exceedingly from his estate being exhausted’ from the Irish mode of tillage.^64^

Young wrote these words, however, at a time when there were a ‘great many sheep’ in Limerick and Tipperary, begging the question of why the large graziers hung on to them if they offered so little profit. Young offered an explanation: ‘The graziers are many of them rich, but generally speaking, not so much from the immediate profit, as from advantageous leases’. Just as we saw for Meath, old leases offered much lower rents to the large graziers and middlemen who had taken long leases, and they suggest a possible explanation of patterns that Thomas Power describes in sheep grazing in his study of Tipperary.^65^

Tipperary is an interesting case since, as Power tells us, some of the county's sheep graziers were engaged in fattening sheep for the Cork and Dublin markets and could hope to make a considerable profit from doing so.^66^ But he notes that ‘the main commercial item from sheep was their wool which went to serve the domestic woollen industry in the county’. Average land rents in Tipperary had doubled by the mid-1770s to about 20*s* an acre, then stagnated due to the American Revolution and war, before rising again from 1790 to reach the same average as Meath by 1800. Given the long-term increase in land rents, therefore, we would expect that sheep graziers who relied on wool revenues would face growing economic pressure.^67^

Sure enough, Power explains, ‘[t]here was a contraction in sheep grazing in the county in the last three decades of the century’, notably in places where it had been conducted most intensively, as it gave way to cereal production and cattle fattening. However, ‘the swing to corn was mainly among small or medium sized farmers and not among the large graziers’, leading to a ‘two-tiered’ agricultural system in Tipperary with ‘landowners and large head tenants’ engaged in pastoral activity while small holders took advantage of bounties in converting land to cereal production.^68^ If Young was right, then the large Tipperary graziers took advantage of older leases to pay lower rents or were landlords who paid no rents at all; for sheep farmers who paid higher rents, in contrast, wool growing would have become less viable as an economic proposition.

More generally, we would expect to see variations in sheep grazing across Ireland depending on the geographical location and economic circumstances of sheep graziers. Still, on the basis of the previous analysis of the profits from sheep grazing and given the limits of the Irish mutton market, we would expect a significant decline in the number of sheep grazing in Ireland in the last quarter of the eighteenth century. In his *Statistical Observations Relative to the County of Kilkenny made in the Years 1800 & 1801*, William Tighe shows how low sheep numbers could go even in a county where the average rent was lower than in Tipperary. As Tighe notes: ‘the average rent of the county paid by the immediate occupier may be about 22*s*. an acre’ and his survey includes references to the suitability of certain parts of the county for sheep and local people's memories of ‘excellent sheep walks’.^69^ Nevertheless, Tighe reports that there were only 45,000 sheep in Kilkenny by 1800, compared to 100,191 people, in a rare example of country-wide statistics.

That implies that Kilkenny grew only 1.8 lbs of wool per inhabitant, which compares with an adjusted estimate of 6.56 lbs per person in Petty's analysis.^70^ But that evidence pertains only to one county, begging the question of whether we can offer a general perspective on the change in Ireland's sheep grazing and wool growing, especially for our focal period from 1780 to 1800. When the Irish Government was asked in 1778 about the number of sheep in the country by a ‘friend of Ireland’ in the British Parliament, the response emphasised the lack of direct evidence on the number of sheep in Ireland.^71^ In fact, counting sheep and wool based on direct observation was rare in Europe in the eighteenth century, so estimates everywhere were derived from indirect methods. Ireland was no exception and in an online Appendix on ‘Counting Irish Sheep’, I review the methods used from William Petty in 1672 to James Laffan in 1786 to generate revised estimates for sheep grazing and wool growing in Ireland.

The results offer some support for revisionists in suggesting that the overall quantities of wool grown in Ireland by the mid-1770s had rebounded to similar levels of total wool production as in Petty's time but much lower levels if calculated in per capita terms. More important here is that the estimates suggest a dramatic decline of between 35 and 40 per cent in sheep grazing and wool growing in Ireland between 1775 and 1800. More research is required but for now these estimates seem to confirm the effects of the political economy pursued by the Kingdom of Ireland in its final decades in discounting of sheep and wool. In this regard, Ireland offers a stark contrast to the parallel efforts in several European states including France and Saxony to promote the improvement of their sheep and wool. But perhaps the most striking contrast is with the long-standing political economy of wool of Ireland's sister kingdom, begging the important question of what changed when Ireland entered a union with Great Britain?

## Defining Ireland's Role in a Union of Wool, 1799–1824

By late 1798, it was apparent that the British Government would propose a union and that John Foster would become its most formidable opponent as Speaker of the Irish House of Commons. Growing concern about French invasion, a major rebellion in Ireland, and the soaring fiscal burden of the British Empire encouraged Pitt to sketch out his plan for union to the British House of Commons on 31 January 1799. In a matter of months, Foster presented a frontal attack on Pitt's scheme to the Irish House of Commons, arguing that the Union would cost Ireland its political autonomy but without any commercial advantage.^72^ Ironically, the political figure who had done more than any other to discount wool and woollens in the pursuit of Irish improvement was to invoke their weaknesses to justify his fierce opposition to a union. In doing so, Foster unwittingly set in motion a series of political manoeuvres that were to lead to a new political economy of wool for Ireland.

Foster invoked the travails of Ireland's wool sector in accusing Britain of only offering ‘the same freedom which Ireland has enjoyed without avail for these 20 years past’ and of failing to remove ‘the disadvantage which now enhances the price of the fabrick in Ireland so as to force her home market to be supplied from Britain, and which enhanced price must equally prevent her from meeting the British at a foreign market’. The ‘disadvantage’ that Foster evoked was the high cost of Irish wool ‘as the great increase of agriculture and of the linen manufacture gave a better profit in land than sheep afforded’. He added that if the minister was to be asked if British wool would be allowed into Ireland, ‘it is obvious what would be the reply’.^73^ Foster had good reason to expect that Ireland would not gain access to British wool given Pitt's commitment to the export ban on wool in negotiating his ‘Irish propositions’ in 1785. Pitt omitted any reference to the ban, moreover, when he secured support in the British Parliament for his broad plan for Union.^74^ In the Irish Parliament, in contrast, Foster's attack contributed to a furore that meant the plan was withdrawn without discussion.

That generated great concern in the Irish and British governments and by the autumn of 1799 a political strategy was in place to undermine Foster's critique. The speaker himself seems to have sown its seed, having suggested to the British Government that ‘the propositions of 1785 should be taken as the basis for the commercial arrangement’ of the Union since they had been seen as reasonable in Ireland.^75^ A prominent advisor to the British Government, Robert Johnston, thought ‘[a]dopting the propositions’ made sense since it would ‘pin the Speaker on his own argument’.^76^ Thus, when the Chief Secretary of Ireland, Lord Castlereagh, presented a detailed plan for union to the Irish Parliament on 5th February 1800, he emphasised that it was similar to the ‘commercial propositions in 1785, which you, Mr. Speaker, supported with such ability’. There was, however, a crucial difference: ‘[b]y the treaty of 1785, the exports of British wool remained prohibited; it [*sic*] is here offered to the Irish manufacturers’.^77^

Castlereagh's intervention gave Pitt's government a rhetorical advantage in front of the Irish Parliament and he seems to have truly believed that ‘the most important advantage to Ireland will be getting wool from England’, allowing it ‘the full recovery of the means of prosecuting the woollen trade’.^78^ But later that year, there were signs that the ‘most important advantage to Ireland’ was slipping away. In evidence before the Irish House of Commons, the Irish yarn merchant, Joshua Pim, explained that: ‘[t]here was formerly great difference in the prices of wool in England and Ireland – they were much cheaper in England than in Ireland’ and ‘[i]t was then a great object to the Irish manufacturer to get share of the English wool’. He added, however, ‘that disparity does not now exist’.^79^ It seems likely from Figure 3, that it was a sharp rise in British wool prices, rather than a decline in Irish prices, that closed the gap.^80^

By early 1800, however, the British government was concerned about potential opposition to Pitt's Union in the British Parliament, stirred up by the country's wool trade with rumours that Foster had helped to stoke its concern.^81^ The British wool trade's petitions highlighted the injustice of an arrangement that would give Ireland free access to British wool while maintaining Irish tariffs on woollen and worsted imports.^82^ They predicted that wool prices would rise further once the Irish could buy English wool, possibly encouraging British capital to move to Ireland to manufacture English wool there given the lure of lower wages and taxes. A vigorous political debate ensued and culminated with a proposal, presented by Mr Wilberforce on behalf of the petitioners, ‘to leave out of the resolution what relates to suffering wool to be exported from this country’ so that ‘the Irish should be allowed to work up the wool which they themselves grow’.^83^

Castlereagh had warned that any such change would undermine the support he had shored up for union in Ireland since it would revive Foster's claim that the plan offered no commercial advantages to Ireland.^84^ But for the first time in more than a century the British woollen trade was to lose a political battle over the export ban. The British Chancellor of the Exchequer dismissed claims that high prices of Irish wool would induce large purchases of English wool, arguing that prices had already converged or would soon converge.^85^

The Union was accomplished in December 1800, and it took effect on 1 January 1801 bringing a major change in Ireland's political economy of wool. Although Irish economic historians have largely focussed on the protective duties for Irish woollen manufactures that the Union allowed, much more significant was the alteration in the political rules that governed the raw wool trade between the two islands. Ireland had long enjoyed the right to export its raw wool, but it was the loosening of the export ban on British wool to allow it to be sold in Ireland that was unprecedented.^86^ Unexpectedly, it motivated political elites in Ireland to restore sheep and wool as a basis for Irish improvement until a gloomier outlook re-asserted itself from the end of the Napoleonic wars.

### Ireland's Sheepish Enlightenment

**Figure 8.**
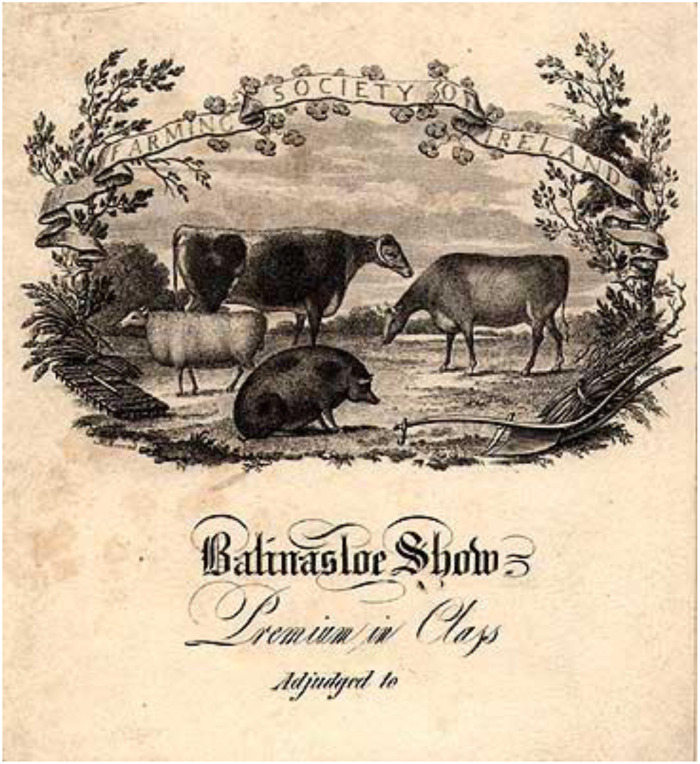
Award for a Farming Society of Ireland premium. *Source*: Engraving by Henry Brocas, National Library of Ireland, PD 2177 (TX) 3, digital image at: https://catalogue.nli.ie/Record/vtls000036729.

There is a further irony in the fact that John Foster, who had fashioned himself as the enemy of Irish woollen manufactures and sheep grazing when he was at the height of his political powers, re-fashioned himself as a promoter of Irish sheep and wool after the Union. At his initiative, the Farming Society of Ireland was established in March 1800, under the patronage of the Dublin Society, with the initial grant for its annual funding secured by Foster during the negotiation of the Act of Union.^87^ Its objective was ‘to improve the agriculture and livestock of the kingdom, and to encourage the best modes of breeding, upon the plan of societies in different parts of Great Britain’ by offering premiums for the best animals presented at Irish fairs (Figure 8).^88^

**Figure 9.**
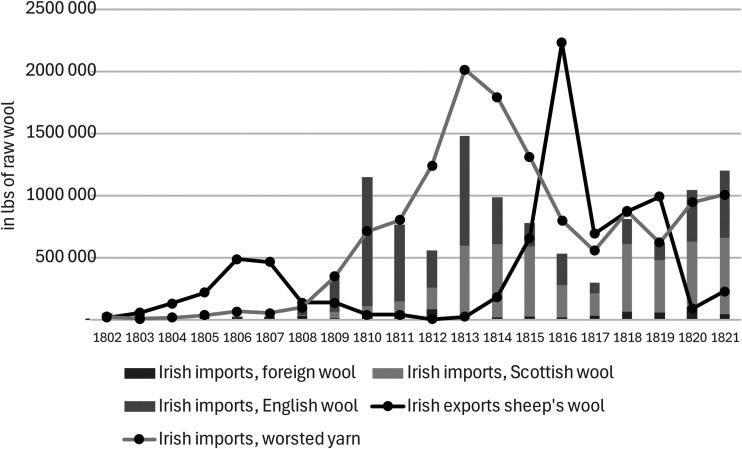
Irish imports and exports of wool and yarn (lbs of raw wool). *Note*: Yarn converted at twenty-five per cent reduction. *Source*: House of Commons, *Fourth Report of the Commissioners of Inquiry into the Collection & Management of the Revenue arising in Ireland*, 1822, Appendix 8, 51.

**Figure 10.**
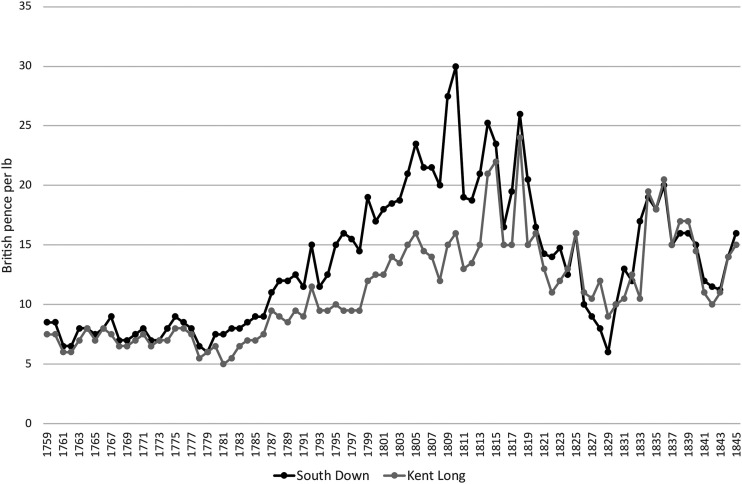
British short and long wool prices. *Source*: P. Deane & B. R. Mitchell, *Abstract of British Historical Statistics* (New York, 1962), 494–5.

The Farming Society's initiatives regarding sheep responded to the dynamics of the United Kingdom's wool market. Contrary to the British wool trade's expectations, as Figure 9 shows, no English wools were imported to Ireland in the years following the Union. Nevertheless, their prices soared due to the expanding demands of British woollen and worsted manufactures. As we have seen, there was a bias towards long wool among Irish sheep graziers and the sharp increase in its price in Britain between 1799 and 1805 induced a brief surge in exports of Irish wool until prices took a less favourable turn (Figure 10). The prices of short wool, in contrast, followed a more favourable and sustained upward trajectory. In pursuit of its belated sheep enlightenment in Ireland, therefore, the Farming Society sought to reverse the established bias among Irish commercial sheep graziers by promoting the growth of short wool.

Ireland already had its own short-wool breeds such as the Wicklow sheep and other sheep bred in mountainous areas. But the Farming Society began by promoting the South Down from England due to the economic potential of its meat and wool. As *Saunders's News Letter* reported, the South Down sheep can be introduced on ‘light grounds and mountainous districts’ and ‘are singularly calculated for travelling when fat from the most remote parts to the metropolis; the excellence and high flavour of their flesh is very generally admitted, and their wool is likely to be very beneficial to the manufacture of fine cloth in Ireland’.^89^ As the price of short wool continued to rise on the British market, the *Dublin Evening Post* reported that ‘[t]he friends to South Down sheep having greatly benefited by the price of their clothing wool, wish to increase its quantity, and are preparing to try whether that object can be attained by a cross with the Merino, without materially injuring the carcase’.^90^

Emphasis on the ‘carcase’ highlights the appeal of the Farming Society's ‘complete’ South Down sheep for elite sheep graziers with access to the country's meat markets. It is true that the Irish mutton market was more unstable from 1801 to 1813 than in the 1790s, as prices fell sharply after the Union but then rose to new heights by the early teens, the number of sheep sold at Ballinasloe fluctuating accordingly. On average, however, the mutton market proved even more appealing to Ireland's elite graziers in the early 1800s than the 1790s, with 30 per cent more on average being paid for mutton and nearly ten per cent more for sheep.^91^ Exorbitant prices for short wool completed the favourable economic conditions for Ireland's sheepish enlightenment and, as prices for the finest short wools reached unprecedented levels, the Farming Society sought to revive interest in Hibernian Merinos, decades after Conyngham's pioneering efforts.

There is no doubting the Farming Society's enthusiasm for promoting short wool sheep and graziers came forward at the Ballinasloe fair to accept the premiums it offered. The highest quality short wools were sold in limited quantities at dedicated auctions organised by the Farming Society.^92^ But as Edward Wakefield observed in 1812: ‘[t]o persons in England acquainted with the wool trade’, the high prices at the Farming Society's wool auctions ‘must appear very extraordinary’.^93^ Despite these high prices, the quantity of South Down wool grown in Ireland was limited, as Foster admitted in a letter to Sheffield in late 1811: ‘[t]he South Down is growing every year a great favourite, but our great object for years to come must be an increase of the quantity of wool’.^94^

In the years to come, however, the economic incentives to increase the quantity of short wool grown in Ireland were undermined by the downward, albeit volatile, trajectory of its price. For graziers close enough to Dublin or Cork to sell fresh mutton, they could fall back on revenues from meat especially given the virtues of the ‘complete’ South Down. Following the Napoleonic wars, however, mutton prices followed a sharp downward course interrupted only by a brief spike in 1817–18. In the south of the country, they fell back to levels not seen since the late 1780s, with sales of sheep at Ballinasloe falling below 60,000 in 1820.^95^ Beyond the reach of the meat markets, the crucial parameter was the price of wool and here a major reversal was underway as well, as short wool lost ground and the price of long wool soared.

**Table 2.** Largest Woollen Manufacturers in Ireland, 1821.

Name	Sales IRL £	Number employed	Capital/employee IRL £
Houghton, Jeremiah	60,000	513	117
Willans, O & Sons	45,000	277	108
Burke, William	30,000	196	153
Irwin, William	22,500	150	53
Nowlan & Shaw	20,000	160	125
Drumgoole, Christopher	17,620	240	67
Murray, John	16,000	126	48
Scott, Kenny	15,000	150	53
% of total	42	30	45
All others	58%	70%	55% Avg. K/L = £47

*Source*: Evidence of Mr J. Houghton, 17 October 1821, House of Commons, *Fourth Report of the Commissioners of Inquiry into the Collection & Management of the Revenue arising in Ireland*, 1822, Appendix 23, pp. 186–7.

The effects on Irish sheep grazing were dramatic, as prominent Irish woollen manufacturer, William Burke, explained: ‘middling clothing wools are at present the only sort that is wanting, the Irish wool being, from the great demand for combing wool for the English market for the years 1815, 1816, 1817, and 1818, so much sought after, that the graziers in all parts of Ireland have turned their whole attention to the growth of this kind of wool’. But the implications were disastrous: ‘In the short period of about six years, the wools of Ireland have been much deteriorated, being very bad clothing and worse combing; our English friends have, in consequence, deserted us entirely’. Sure enough, as Figure 9 shows, Irish exports of raw wool climbed above 2 million lbs in 1816 only to collapse to 200,000 lbs by 1821 when Irish trade statistics end. The reasons for the collapse were clear enough, as Burke explained: ‘Scotch and English friends have better wool to sell us at cheaper prices’.^96^

Despite the high profile of the Farming Society of Ireland and its efforts to promote Irish sheep, therefore, the sheepish enlightenment in Ireland was an ignominious failure. Another prominent woollen manufacturer, William Willans, estimated that, ‘[t]he wool grown in Ireland in 1821’ amounted to ‘5000 bags, of 50 stones each’, representing 3.5 million lbs of wool in total. If we assume the average fleece weighed 4 lb, that was equivalent to the wool of 875,000 sheep, implying the each Irish person at the time consumed only 0.5 lb of Irish wool or the growth of a mere 13 per cent of an Irish sheep.^97^ The contrast with Great Britain is startling with consumption of British wool estimated at 7.6 lb per capita in 1821, representing the growth of nearly two sheep (with 4 lb fleeces) per person.^98^

By that time, domestic wool still represented the vast majority of wool consumed in Britain but that was no longer the case in Ireland. In 1821, just as Burke suggested, Ireland imported 1.2 million lbs of raw wool in roughly equal quantities from England and Scotland. In addition, wool came into Ireland in manufactured form as imports of woollen and worsted cloth. Finally, there was a reversal in the worsted yarn trade as Ireland imported large amounts of wool in machine-produced imports from Britain. Overall, Ireland consumed about 9.6 million lbs of raw wool in 1821, representing 1.41 lb per capita, of which less than forty per cent came from Irish sheep.^99^

According to Burke, the consequences were already apparent in the visible lack of sheep in large parts of the Irish countryside, noting that he had

travelled through the Galtees, and the beautiful pasture mountains of Waterford, Cork, Kerry, Tipperary, Mayo, and Roscommon – I could find a few scattered sheep and some goats in a ride of several miles; a kind of smoking volcano, called a cabin, the residence of man and beast, if any were so fortunate to have a pig….

And he warned that ‘[i]f the legislature takes off the small protection we now enjoy, they ought at the same time annex a clause to destroy all the sheep in Ireland, as they would then become an unprofitable animal, without having a market, either at home or in England, to dispose of their wool’. His remark invites us to complete this sheepish account of Ireland's changing role in an imperial age with a brief chapter on the resurgence and decline of the Irish woollen sector following the Union.

### Wool as an ‘Unnatural’ Industry for Ireland, 1801–24

In evidence given at the time of the Union, it was clear that representatives of the British woollen trade were little concerned about any possible competitive threat from Irish woollen manufactures. That makes sense given what we have seen of their condition by the late eighteenth century. Greater credibility was given to the prospect that British capital might be lured to Ireland by low labour costs and taxes to manufacture wool for sale in Ireland's protected market and for export to Britain. In the years following the Union, however, there seemed little sign of any great change in the Irish woollen sector.

**Figure 11.**
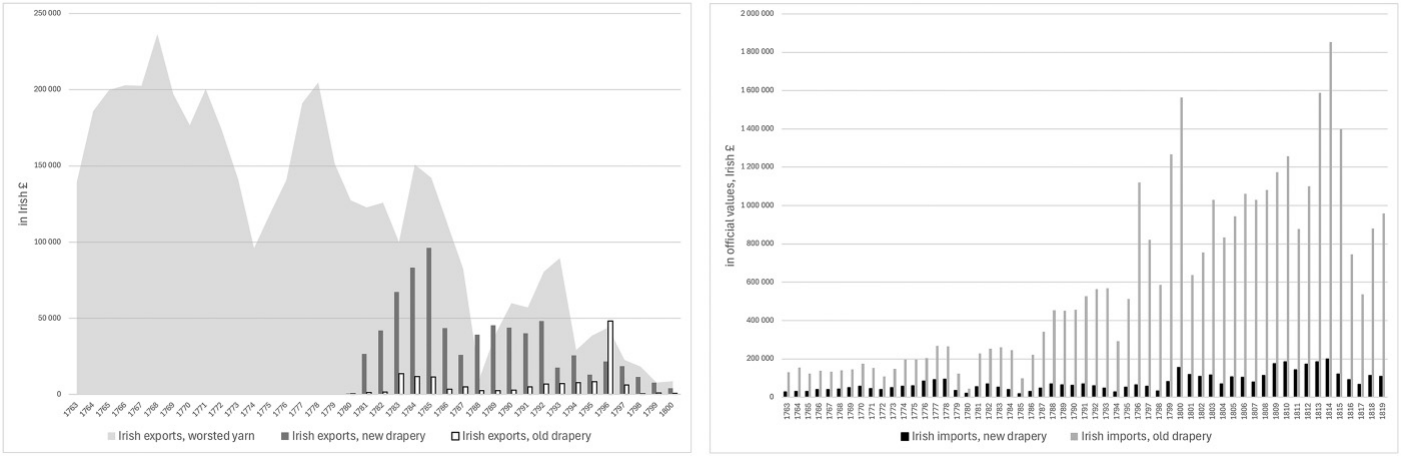
Irish trade in woollen and worsted goods, 1763–1819. *Source*: *Ledgers of Imports and Exports, Ireland*, CUST 15, various issues, National Archives, Kew Gardens, London.

There was a limited recovery in exports of Irish worsted yarn in the early years of the century, but it soon faded, and Irish woollen and worsted exports continued their downward movement. Even more dramatically, imports of British goods rose sharply from the Union to the end of the Napoleonic wars. Despite occasional signs of commercial vibrancy, Irish woollen manufactures continued to depend heavily on military demand. Indeed, the weakness of the commercial part of the Irish woollen sector stimulated great concern in the Farming Society of Ireland as an obstacle to its plans for improving Irish wool and sheep (Figure 11).

It introduced manufacturing premiums to show how better Irish wool could be employed in the manufacture of cloth. The initiative proved insufficient, as is clear from the following announcement in 1810:

From the present distressed situation of the Woollen Manufacturers, the Committee have thought it expedient to postpone the Sales of Fine Wool which should have taken place on the 16th inst. to a future day… and consequently the Shew of Broad Cloth and Kerseymeres, which should have been on the 26th Sept is likewise postponed.^100^

And it was not just the finer manufactures that suffered at the time, with reports that ‘owing to the present stagnation of the Coarse Woollen Trade, some of the Clothiers are now on their return from the Great Wool Fair of Ballinasloe, without making any purchases’.^101^

Still, even if manufacturing had decreased in some places, Edward Wakefield reported in 1812 that it was ‘evidently upon the increase’ in others.^102^ He offered Leinster as an example, where he said that ‘[a] small quantity of broad cloth is manufactured in Dublin, and within these few years, a Yorkshire firm has been driven to Cellbridge [*sic*] in the country of Kildare, where they have established the shearing machinery, which the Yorkshire manufacturers would not permit to be employed in their own county’. Laurence Atkinson and James Haughton from Yorkshire had established a woollen business in Celbridge in 1805.^103^ They were not alone with a series of mills opened or extended around the same time by English and some Irish capitalists to conduct woollen manufacturing in Ireland.

The largest ones are shown in Table 2 based on evidence given in 1821 to a parliamentary enquiry. The Celbridge firm is shown under Jeremiah Houghton's name as the leading mill in Ireland with William Willans and William Burke ranked just behind. For manufacturers of finer woollen cloth like Houghton, wool was imported from England with the rest from Irish mountain sheep; other mills produced coarser cloth from Irish wool for sale to the local population who had relied on home spun and woven goods until then. It is clear from Table 2 that these firms’ significant investment in machinery was a distinctive feature of their operations. As we have seen, Wakefield claimed the relative facility in introducing machinery in Ireland is what encouraged the development of such enterprises.^104^ However, when mill owners were asked for their reasons for establishing operations in Ireland, they emphasised low labour costs.

Quizzed about the basis for his ‘expectations that the price of labour could be reduced to such an extent, as to produce goods that would enter into competition with the English in the foreign market?’, Jeremiah Houghton replied that ‘[w]e thought living would have been much cheaper here than we afterwards found it’ and noted that he had induced English workmen to Ireland on that basis. But he explained that

[t]hey found on being transplanted into this country, that the additional price of fuel, rent, and a variety of other considerations, counterbalanced the cheapness offered. That description of people pay higher rents here than they do in England: my weavers, many of them, pay from two to three or four shillings a week.

It is notable that many of these men had chosen to locate their large mills ‘in Dublin or its vicinity’ where the rents that drew their men's complaints fell in a range from £5.2 to £10.4 a year.^105^ That represented a major increase on the ‘high rents’ of £3 per annum that Luke Gardiner had reported for the city of Dublin in 1778.

Just how far Ireland had come from a time when even men like John Foster could speak of wool as the country's ‘staple’ or ‘natural manufacture’ is evident in the parliamentary committee's suggestion that Ireland's largest woollen mills were engaged in an ‘unnatural manufacture’ in which they had no distinctive advantage. Unnatural or not, they did not survive for much longer. In evidence given in 1838, William Willans argued that the scale of the industry had already been ‘estimated too high’ in 1821 being based on what woollen manufacturers ‘could produce if fully employed, rather than the amount which they actually did produce’. But his main emphasis was on the industry's subsequent decline: in Dublin, the manufacture had ‘fallen off more than one-half’, woollen manufactures in the districts of Cork, Kilkenny, Moate and Carrick-on-Suir were hardly ten per cent of the ‘overrated’ activity reported for 1821. He added that a different type of woollen manufacture, in which machinery played no role, had featured in the 1821 report. It was the flannel trade of Wicklow and Wexford and at the time, as William Burke had noted: ‘[t]he tenantry of nearly the entire county of Wicklow pay their rents with mountain wool… and a great manufacture is carried on in flannels, frizes, cadows’. In 1838, in contrast, Willans reported that: ‘it is now almost extinct as from the best information I can procure it does not at present amount to £500 per annum’.^106^

Willans did not blame the removal of protective duties in 1825 for the reduced condition of Ireland's woollen sector. He pointed instead to a great influx of cut-price manufactures from Britain that crushed large numbers of Irish manufacturers. It followed a dramatic collapse in British prices when the wool market was liberalised, which induced British manufacturers to discount their goods heavily. For similar reasons, an earlier drop in the price of Merino wool on the British market had brought an end to the Merino factory in Kilkenny, one of Ireland's most ambitious experiments in the woollen sector.^107^

With the virtual demise of Ireland's woollen manufactures, we conclude this essay very close to where we began. After all, it was Willans’ testimony that offered L. M. Cullen the basis for his startling observations about the reduced condition of Ireland's woollen sector.^108^ But Cullen does not report another of Willans's observations that: ‘[a]s respects the consumption in Ireland of woollen goods, it is much below that of an equal population’. That fact is anticipated above in the difference in wool consumption per capita in Ireland and Britain. Willans offered further evidence from 1,200 dealers across Ireland to estimate that the average Irish person spent only 4s a head on woollen goods, which he estimated as only 20 per cent of the £1 spent by the average person in England and Scotland.

Though the Irish consumed relatively little wool by the late 1830s, that did not imply the end of the Irish sheep. Contrary to Burke's expectation that sheep numbers would collapse with the demise of Irish woollen manufactures, Willans reported that there had been some increase in the growth of Irish wool to 4.9 million lbs by 1837, representing the growth of 1.2 million sheep with 4 lb fleeces. Once again, if we follow the money, we find it partly in a recovery in mutton prices and especially in the major rebound in wool prices in the British Empire. It meant, as Willans explained, that ‘the great bulk’ of Irish wool, despite its coarse quality, was exported to England, ‘perhaps two-thirds, or even more’.^109^ The course of wool and mutton prices in the ensuing years would lead us to expect some further expansion in Irish sheep numbers, especially since as Peter Solar explains, the introduction of steamships on the Irish Sea made the British mutton market accessible to Irish sheep graziers.^110^ The estimate of 2.1 million sheep in Ireland from the 1841 Census would seem to confirm that trend. Even so, that still meant there were only 0.26 sheep per head of population in Ireland by 1841, a far cry from the 1.5 sheep per capita that its neighbouring island could still boast even after decades of heightened industrialisation.^111^

## Conclusion

In recounting the history of the long demise of Irish sheep and wool, this essay shows the value of integrating political economy and economic history. The approach overcomes a major gap that exists between these fields and that impedes our understanding of important interactions between the political and intellectual history of economic ideas and historical patterns of economic life. As the essay shows, such a compartmentalised approach is highly problematic for Irish wool given its importance in Ireland's internal political dynamics and its changing relationship with Britain, as well as the profound impact of these politics on the economic history of Irish sheep and wool.

The essay shows that the weak commercial standing of the Kingdom of Ireland's wool sector on the eve of the Act of Union had political as much as economic origins. Despite Ireland's enhanced political autonomy in the closing decades of the eighteenth century, influential members of the Irish political elite refused to take steps to redress the unequal access of the sister kingdoms to their respective markets for woollens and worsteds. Worse still, Irish political leaders allowed British manufacturers access to the Irish market on relatively easy terms without insisting they share their access to cheaper and better wool with their Irish counterparts. As a result, they turned the Irish woolen sector into an industry vulnerable to British competition.

Even if Irish political elites were wary of confronting their sister kingdom to rewrite the political rules of wool exchange, there were steps they could have taken at home to reduce the cost and improve the quality of Irish raw wool. Indeed, for most of the eighteenth century, they had responded to British restrictions on Irish wool and woollens with considerable effort and expense to promote the import of high-quality flax seeds and the extension of flax cultivation. Yet, even when these restrictions were lifted, and Irish political elites had the option of promoting wool growing, they chose active discouragement instead. Their politics of agricultural improvement encouraged an extension of tillage and an increase in rentals that undermined the economic viability of wool growing for many graziers. The problem was reinforced by a system of landholding in which landowners, larger tenants, and middlemen enjoyed access to land at lower costs than small tenants.

In waiting for the Union to launch a sheepish enlightenment, Irish politicians lost whatever latitude they had enjoyed to shape the future of Irish wool growing and manufacture. Both in agriculture and manufacturing, Irish sheep and wool had no advantage in competition with their British counterparts. Irish wool of poor quality could be sold in the British market but only at discounted prices and the Irish manufacture of woollens and worsteds had so little distinction that it could be cast as an ‘unnatural’ manufacture. British workmen were shocked at the cost of living in Ireland, not least the high rents they were expected to pay, and proved unwilling to accept the compromises in accommodation, diet and clothing that Irish working people were forced to tolerate if they stayed at home.

That the essay recounts a history of sheep and wool that hints at broader implications can be seen if we return to Lavoisier's characterisation of sheep as the beating heart of a powerful economic and environmental system. His observations challenge the complacency of a man like Sheffield who blamed low levels of mutton consumption in Ireland on the indolence of its woollen manufacturers. Lavoisier's analysis points, instead, to the need to think systemically in addressing the question of why the grazing of sheep, the manufacture and diffusion of woollen manufactures and the consumption of mutton were so high in Britain and so low in Ireland by the late 1830s? In pursuing such a systemic approach, moreover, Lavoisier invites us to broaden the scope of our thinking to include the ecological as well as the economic implications of a sheep-based system. Such ecological implications found expression in the late eighteenth and early nineteenth centuries in Ireland in growing concerns about a shortage of fertiliser. A series of letters by an absentee Irish landlord captured the spirit of these concerns in 1824 in exhorting the promotion of sheep to provide ‘manure for our lands’ rather than exhausting them with flax.^112^

## Notes

1 *Exposé de la Situation de l’Empire présenté au Corps Législatif dans sa Séance du 25 Février 1813 Par S. Exc. Le Comte de Montalivet, Ministre de L’Intérieur* (Paris, 1813), pp. 13–14; J. Holroyd, Lord of Sheffield, *On the Trade in Wool and Woollens Including an Exposition of the Commercial Situation of the British Empire* (London, 1813), pp. 8–11.

2 G. Riello, ‘Nature, Production and Regulation in Eighteenth-Century Britain and France: The Case of the Leather Industry’, *Historical Research* 81:211 (2007), 84.

3 Author's calculations based on 21 January 1788, Donnington near Newbury Berks, Appendix III in H. B. Carter (ed.), *The Sheep and Wool Correspondence of Sir Joseph Banks 1781–1820* (London, 1979), pp. 530–1.

4 A. Lavoisier, ‘Sur l’Agriculture et le Commerce de l’Orléanais’, *Mémoire à l’Assemblée provinciale de l’Orléanais*, 1788 in G. Schelle et E. Grimaux, *Lavoisier : Statistique agricole et projet de* réformes (Paris, 1894), pp. 40–1, author's translation.

5 J. Hoppit, *Britain's Political Economies: Parliament and Economic Life, 1660–1800* (Cambridge, UK, 2017), pp. 216–48.

6 W. Petty, *The Political Anatomy of Ireland: With the Establishment for that Kingdom When the Late Duke of Ormond was the Lord Lieutenant* (London, 1791) [written c. 1672], p.81.

7 A. Murray, *A History of the Commercial and Financial Relations between England and Ireland, from the Period of the Restoration* (London, 1903), pp. 62–5.

8 L. Clarkson, ‘The Carrick-On-Suir Woollen Industry in the Eighteenth Century’, *Irish Economic and Social History* 16 (1989), 23.

9 For early elements of a revisionist turn, see H. F. Kearney, ‘The Political Background to English Mercantilism, 1695–1700’, *Economic History Review*, 11:3 (1959), 484–96; P. Kelly, ‘The Irish Woollen Export Prohibition Act of 1699: Kearney Re-Visited’, *Irish Economic and Social History*, 7 (1980), 22–44. Ensuing contributions include L. M. Cullen, *Anglo–Irish Trade*, 1660–1800 (New York, 1968); Clarkson, ‘Carrick’; A. Bielenberg, *Cork's Industrial Revolution, 1780–1880* (Cork, 1991); and D. Dickson, *Old World Colony: Cork and South Munster, 1630–1830* (Cork, 2005).

10 On ‘the destruction of the Irish cattle trade’ as marking ‘a new intensification of English control over Irish economic life’, see C. A. Edie, ‘The Irish Cattle Bills: A Study in Restoration Politics’, *Transactions of the American Philosophical Society*, 60:2 (1970), 1–66; on the related theme for wool and woollens, see D. O’Hearn, *The Atlantic Economy: Britain, the US and Ireland* (Manchester, UK, 2001), pp. 63–7.

11 P. M. Austin, ‘The Agricultural Statistics of the 1841 Census of Ireland. A Critical Review,’ *Economic History Review*, 18:2 (1965), 381.

12 L. M. Cullen, *An Economic History of Ireland since 1660*, 2nd ed. (London, 1987), p. 106.

13 J. Stafford, ‘Desolation and Abundance: Poverty and the Irish Landscape, c. 1720–1820’, in N. O’Flaherty and R. J. W. Mills (eds), *Ideas of Poverty in the Age of Enlightenment* (Manchester, 2024), pp. 188–211.

14 G. Brownlow, C. Cox and E. McLaughlin, ‘Irish Economic & Social History; Publication Trends’, *Irish Economic and Social History*, 51:1 (2024), 1–9.

15 M. Nix, *Norwich Textiles: A Global Story, 1750–1840* (Norwich, UK, 2023).

16 William Conyngham Burton to Joseph Banks, 28 June 1788, in H. B. Carter, *Correspondence*, p. 155.

17 For a comprehensive discussion, see M. O'Sullivan, ‘Ireland's Role in British Colonial Capitalism: “Men of Capitals” and Pitt's Irish Proposals, 1784–1785’, *Business History Review*, 98:1 (2024), 119–63.

18 Opinion of John Foster, 7th July 1779, reprinted in G. O’Brien, ‘The Irish Free Trade Agitation of 1779’, *The English Historical Review*, 39:153 (1924), 95, 100.

19 On the role of other imperial legislation in inhibiting Irish trade, notably the Navigation Acts, see the important debate on this issue, led by the Irish Chancellor of the Exchequer, John Foster, in 1784–1785 (O'Sullivan, ‘Ireland's role’).

20 The exception was made in 1739 to take effect in 1740 (Cullen, *Economic History*, pp. 3–4).

21 Nix, *Norwich Textiles*, pp. 112–27.

22 Based on *Ledgers of Imports and Exports, Ireland*, CUST 15, various issues, National Archives, Kew Gardens, London, allowing a reduction of twenty-five per cent in the weight of raw wool transformed into yarn.

23 *Ibid*.

24 L. Gardiner, *Report from the Committee Appointed to Enquire into the State of the Manufactures of this Kingdom, and What may be the Necessary for the Improvement Thereof, and also into the Quantity and Value of the Exports and Imports of Ireland*, 5 Mar. 1784, in *Journals of the House of Commons of the Kingdom of Ireland* (Dublin, 1784), Appendix, cxxxv–ccvi; for a discussion, see O'Sullivan, ‘Ireland's Role’, pp. 26–32.

25 Nix, *Norwich Textiles*, pp. 105–12, especially p. 111.

26 P. Solar, ‘Growth and Distribution in Irish Agriculture Before the Famine’ (Ph.D. dissertation, Stanford University, 1987); see also L. Kennedy and P. M. Solar, *Irish Agriculture: A Price History* (Dublin, 2007).

27 Testimony of Mr J. Pim, ‘Report from the Grand Committee of Trade’, 6 March 1782, in *Journals of the House of Commons of the Kingdom of Ireland*, Parliament, House of Commons, Kingdom of Ireland (Dublin, 1782), Appendix, pp. cccclxxxix–ccccxc.

28 The memorandum noted that ‘[a] Broad Cloth Weaver cannot carry on his trade without two looms, for which he will, at least, pay in the City £3 a year. Whereas in the Country at some distance from the Metropolis, he will have a comfortable Cabbin, and three or four acres of Ground at the same expence’. It added that in England ‘the greatest part of the Woollen Manufacture is carried on at a great distance from the Metropolis’ (‘Copy Answer to Lord Nugents letter on the Woollen Trade’ by L. G. Esq., 1778, written in James Corry's handwriting and attributed to Luke Gardiner, Public Record Office of Northern Ireland (PRONI), Belfast, Northern Ireland, D 562/8674.

29 Testimony of Richard McCormick, Gardiner, *Report*, p. cxxxvi.

30 Mr Nixon, *Observations on the Growth of Wool*, reproduced in Joseph Archer, *Statistical Survey of the County Dublin: With Observations on the Means of Improvement; Draw Up for the Consideration, and by Order of the Dublin Society* (Dublin, 1801), p. 169. Archer reports that the memoir was sent to him by Nixon, a manufacturer from Chapelizod, that it was mostly written in 1784 and addressed to the Right Hon. John Foster (p. 162).

31 John Parnell to Thomas Pelham, 31 December 1784, Protecting Duties, Bolton Manuscripts, MS 15,846/(2), National Library of Ireland (NLI), Dublin, Ireland.

32 *Ibid*.; John Foster to Sheffield, 23 December 1786, PRONI T2965/70.

33 In presenting economic incentives for different types of agriculture in such pragmatic terms, Foster concealed the political decisions that shaped agricultural prices and bounties. On the importance of these influences for British textiles, as well as their impact on interconnections between England, Ireland and Scotland, see respectively, P. O'Brien, T. Griffiths and P. Hunt, ‘Political Components of the Industrial Revolution: Parliament and the English Cotton Textile Industry, 1660–1774’, *Economic History Review*, 44:3 (1991), 395–423; T. Griffiths, P. Hunt and P. O'Brien, ‘Scottish, Irish, and Imperial Connections: Parliament, the Three Kingdoms, and the Mechanization of Cotton Spinning in Eighteenth-Century Britain’, *Economic History Review*, 61:3 (2008), 625–50. My thanks to Eoin McLaughlin for highlighting this link for me.

34 For a general discussion of Dublin's master manufacturers and journeymen, including their religious affiliations, see J. Hill, *From Patriots to Unionists Dublin Civic Politics and Irish Protestant Patriotism, 1660–1840* (Oxford, 1997), pp. 133–70.

35 O'Sullivan, ‘Ireland's Role’.

36 John Foster, *Report on Bounties, from the Committee Appointed to Enquire into the Expenditure of £15,000 Granted Last Session*, 12 February 1785, in *Journals of the House of Commons of the Kingdom of Ireland*, vol. 20, 20 January 1785–7 September 1785 (Dublin, 1785), Appendix, cccliii–ccclx.

37 *Ibid*.

38 NLI, A14648, William Pitt to the Duke of Rutland, 6 January 1785.

39 In a file marked ‘Objects Required by Ireland, 4th June 1784’ in Thomas Orde's papers (Bolton Manuscripts, NLI, MS15821(2)).

40 Nixon, *Observations*, pp. 163–4, 175.

41 Mr Ogilvie and Mr Corry respectively in *The Parliamentary Register: or, History of the Proceedings and Debates of the House of Commons of Ireland*, Vol. 7, 1787, pp. 283, 292–3.

42 Nix, *Norwich Textiles*, pp. 113–18, 464–5.

43 The estimates of the number of people employed are close to those furnished for woollen manufacturing in 1792 in John, James Whitelaw and Robert Walsh, *History of the City of Dublin from the Earliest Accounts to the Present Time*, Vol. 2 (Dublin, 1818), p. 983.

44 ‘Petition of the Working Worsted Weavers, the Manufacturers of Bay Yarn, of the City and Liberties of Dublin’, *Parliamentary Register*, Vol. 13, 1793, pp. 449–50.

45 Irish Legislation Database, Queen's University Belfast.

46 Warburton, Whitelaw and Walsh, *Dublin*, Vol. 2, p. 983.

47 Just over thirty-eight per cent of the people arrested compared to much lower shares for other types of worker (T. Murtagh, ‘Hibernian Sans-Culottes? Dublin's Artisans and Radical Politics, 1790–1798’, *La Révolution française: Cahiers de l’Institut d’histoire de la Révolution française*, 11 (2016), 12).

48 A. Bielenberg, ‘Industrial Growth in Ireland, c. 1790–1910’ (Ph.D. thesis, University of London, London School of Economics, 1994), pp. 102–3.

49 Robert Thompson, *Statistical Observations Related to the County of Kilkenny* (Dublin, 1802), pp. 543–8. For a detailed analysis for other woollen manufacturers, especially in the important centre of Carrick-on-Suir, see Clarkson, ‘Carrick’.

50 As prominent Yorkshire manufacturer, Benjamin Gott, explained of Ireland in 1800: ‘there being a larger number of troops in the country than usual, more of the wool of the country is necessarily employing in clothing them, and in finding bedding and blankets’ (Wool Committee, *An Account of the Proceedings of the Merchants, Manufacturers, and Others, concerned in the Wool and Woollen Trade of Great Britain, in their Application to Parliament, that the Laws Respecting the Exportation of Wool Might Not Be Altered, in Arranging the Union with Ireland, but Left to the Wisdom of the Imperial Parliament, if Such a Measure Should Hereafter Appear to be Just and Expedient* (London, 1800), pp. 140–1).

51 J. Holroyd (Sheffield), *Observations on the Manufactures, Trade*, *and Present State of Ireland* (London, 1785). Sheffield's observations appeared in a comprehensive analysis of the Irish economy that he published in 1785 to stir up opposition in Britain to Pitt's Irish commercial propositions.

52 *Ibid*.*,* p. 164.

53 *Ibid*.; see also p. 18.

54 Even well into the nineteenth century, it was believed, as John Maitland observed, that: ‘[t]he carcass of the sheep materially differs from those of cattle or swine, for the fat of it becomes so rancid and ill flavoured, under salting, that it is never (like beef and pork) exported as an article of merchandise’ (J. Maitland, *Observations on the Impolicy of Permitting the Exportation of British Wool, and of Preventing the Free Importation of Foreign Wool* (London, 1818), p. 7).

55 Holroyd, *Observations*, p. 162.

56 Report from the Lords Committees appointed to enquire into the State of Tillage in this Kingdom, Irish House of Lords, Dublin, 1757; A. Young, *Tour in Ireland*, p. 275.

57 K. Connell, ‘The Balance Between Pasture and Arable’, chapter 4 in *The Population of Ireland, 1750–1845* (Oxford, 1950), especially pp. 108–9.

58 Solar, ‘Growth and Distribution’, pp. 59–68; Kennedy and Solar, *Irish Agriculture*, pp. 56–62.

59 See the online Appendix.

60 Even at high prices of wool of 11 to 12*d* per lb, stock rates of five sheep per acre, and fleeces weighing an average of 5 lb, a grazier would generate revenues from wool of about £1 4*s* a year. Tillage generated much higher revenues per acre and when market prices satisfied the conditions for bounties to be paid under Foster's Corn Law, which as Connell notes meant for most years between 1784 and 1800, they offered tillage farmers an additional revenue per acre of between 40 and 57 per cent of a grazier's entire wool revenues.

61 R. Thompson, *Statistical Survey of the County of Meath* (Dublin, 1802), pp. 225–6.

62 *Ibid*.

63 In a succinct way, Thompson captured the crucial distinction between rents at new lettings, whether they were set by landlords or middlemen, and rents established in long leases. As Solar and Hens observe, most studies of land rents per acre in Ireland in the eighteenth century use leases to track rents, which generate much lower estimates than the averages that men like Thompson reported (P. Solar and L. Hens. ‘Land under Pressure: The Value of Irish Land in a Period of Rapid Population Growth, 1730–1844’, *Agricultural History Review*, 61:1 (2013), 40–62.

64 Young, *Tour of Ireland*, pp. 382–3.

65 T. Power, ‘Land, Politics, and Society in Eighteenth Century Tipperary’ (Ph.D. thesis, Trinity College Dublin, June 1987).

66 *Ibid*., pp. 217–18.

67 *Ibid*., p. 47.

68 *Ibid*., pp. 214–15, 233. Nor were such advantageous terms necessarily confined to old leases, given the advantageous ‘resets’ that Power highlights in Tipperary during the years of American turbulence when rents had retreated (*ibid*., p. 107).

69 W. Tighe, *Statistical Observations Relative to the County of Kilkenny Made in the Years 1800 & 1801* (Dublin, 1802), p.19.

70 Based on adjusting Petty's population estimates from 1.1 million (and later 1.3 million) to 1.8 million to take account of historians’ criticisms.

71 PRONI, Belfast, Northern Ireland, D 562/8674.

72 *A Report of the Important Debate in the House of Commons of Ireland on Thursday, 11 April 1799 on the Regency Bill including an Authentic Copy of the Speech of the Right Honorable John Foster* (Dublin, 1799).

73 *Ibid*., p. 72.

74 *The Parliamentary Register: or, History of the Proceedings and Debates of the House of Commons of Great Britain*, Vol. 54, 1799, p. 182.

75 ‘The Speaker's Ideas Concerning a Commercial Arrangement’, no date [late 1799], in C. W. Vane, Marquess of Londonderry (ed.), *Memoir and Correspondence of Viscount Castlereagh* (London, 1848), III, pp. 180–1.

76 *Ibid*., p. 181.

77 *The Speech of the Right Honorable Lord Viscount Castlereagh upon delivery to the House of Commons of Ireland His Excellency the Lord Lieutenant's Message on the Subject of Incorporating Union with Great Britain with the Resolutions; Containing the Terms on which it is Proposed to Carry that Measure into Effect*, 5 February 1800 (Dublin, 1800), pp. 27, 29.

78 Lord Castlereagh, ‘Notes on the Commercial Article (6)’, no date [likely October or November 1799], *ibid*., p. 187.

79 *Report of Evidence taken before the Irish House of Commons on the Legislative Union*, UK, House of Commons Papers, 35 (London, 1833 (517)), 541 fos.

80 We cannot be sure since prices for Irish wool do not seem to have been published in the usual newspapers for 1800.

81 G. C. Bolton, ‘Some British Reactions to the Irish Act of Union’, *Economic History Review*, 18:2 (1965), 367–75.

82 Even Foster grudgingly admitted the ‘liberality’ of the ‘free import of wool from Britain’ and ‘that it was not included in the commercial propositions, of 1785’ (*Speech of the Right Honorable John Foster, Speaker of the House of Commons of Ireland: delivered in committee, on Monday the 17th day of February, 1800* (Dublin, 1800), p. 23.

83 Mr Wilberforce, 1 May 1800, in *Wool and Woollen Trade of Great Britain*, p. 221.

84 Castlereagh to Mr Cooke, 11 April 1800 in *Castlereagh*, 275.

85 Chancellor of the Exchequer, 1 May 1800, *Wool and Woollen Trade of Great Britain*, pp. 233–4.

86 Besides the Irish exception, the export ban on British wool was to remain in place, as Hoppit explains, until it was lifted in 1824.

87 A. P. W. Malcolmson, *John Foster (1740–1828): The Politics of Improvement and Prosperity* (Dublin, 2011), pp. 374–6; for a useful though hagiographic account of its history, see ‘The Farming Society of Ireland' in Warburton et al., *Dublin*, Vol. 2, 960 fos.

88 Archer, *Dublin*, p. 142.

89 *Saunders's News Letter*, 13 October 1802.

90 *Dublin Evening Post,* 12 October 1809.

91 Average prices are based on Solar's Belfast series for mutton since it is the only one that offers a sufficiently complete range of data to make the calculation. An average of just over 72,000 sheep per year was sold at the Ballinasloe fair from 1801 to 1813, nine per cent higher than the 66,000 reported above for the 1790s. There were sharp but temporary peaks of more than 87,000 sheep sold in 1803 and 1806 (see the online Appendix for further details).

92 See, e.g. ‘Annual Sale of Clothing Wool sold in Ireland, July 17, 1809’, printed in Edward Wakefield, *An Account of Ireland, Statistical and Political*, 1 (London, 1812), pp. 713–15.

93 *Ibid*., p. 715.

94 Foster to Sheffield, 24 November 1811, PRONI T3725/38. Hibernian Merinos produced only tiny amounts of wool at this time (Warburton et al., *Dublin*, Vol. 2, p. 963).

95 Solar, ‘Growth and Distribution’, Appendix Table 2.7.

96 *Dublin Weekly Register*, 24 November 1821.

97 Population of Ireland was 6.8 million according to the 1821 Census (Census of Ireland, 1821, *Accounts and Papers*, British Parliamentary Papers, 1824).

98 Testimony of William Willans, 24th May 1838, *General System of Railways for Ireland: Second Report, Appendices and Index by Great Britain. Royal Commission appointed to enquire into the Manner in which Railway Communications can be Most Advantageously Promoted in Ireland* (Dublin, 1838), Appendix A, 8, p. 76. Based on estimates of the domestic wool clip of 110 million lbs and the estimated population of 14.1 million for England, Scotland and Wales according to the 1821 Census (Deane and Mitchell, *Historical*, p. 6).

99 See the online Appendix for details.

100 *Dublin Evening Post*, 12 July 1810.

101 *Saunders's News Letter,* 25 July 1810.

102 He mentioned ‘Midleton, Carrick, and Kilkenny’ as examples of decreasing manufactures (Wakefield, *Account*, pp. 717–18).

103 Wakefield reported that ‘[t]hey import much of their wool from England’ with the rest from Irish mountain sheep (*ibid*., p. 717).

104 House of Commons, *Fourth Report of the Commissioners of Inquiry into the Collection & Management of the Revenue Arising in Ireland*, 1822, Appendix 23, 186–7.

105 The Nowlan and Shaw mill in Kilkenny, the Merino Factory, was deliberately located outside of Dublin to avoid the capital city's supposedly pernicious influence on its working people (S. Curran, ‘The Merino Factory at Ennisnag, Co. Kilkenny’, *Old Kilkenny Review*, 37 (1985), 105–19; W. J. Pilsworth, ‘The Merino Factory: Old County Kilkenny Industry’, *Old Kilkenny Review*, 2 (1948), 26–31.

106 Willans, *General System*, p. 76.

107 The Merino factory went bankrupt in 1821 (Pilsworth, ‘Merino Factory’, 31).

108 Cullen, *Economic History*, p. 106.

109 Sold at an average price of 12.9*d* per lb based on Willans's estimates, compared to 15*d* per lb for Southdown and Kent Long wools in 1837.

110 P. Solar, ‘Shipping and Economic Development in Nineteenth-Century Ireland', *Economic History Review* 59:4 (2006), 732.

111 For Irish sheep numbers, see Austin, ‘Agricultural Statistics’, 381; for a breakdown of sheep in the United Kingdom by country, see P. Deane and W. A. Cole, *British Economic Growth, 1688–1959: Trends and Structure* (Cambridge, UK, 1962), p. 195.

112 *Turnips, Sheep, Wool, and Prosperity: versus Flax, Potatoes, Mud Hovels and Poverty, being a Series of Letters, in which the Soundness of the Policy of Extending the Growth of Wool and Permitting its Free Export [are] Clearly Demonstrated*, by an Irish landlord (Bristol & London, 1824), Haliday Collection, Royal Irish Academy, Dublin, Ireland.

